# The impact of pharmacokinetic gene profiles across human cancers

**DOI:** 10.1186/s12885-018-4345-2

**Published:** 2018-05-21

**Authors:** Michael T. Zimmermann, Terry M. Therneau, Jean-Pierre A. Kocher

**Affiliations:** 1 Division of Biomedical Statistics and Informatics, Department of Health Sciences Research, College of Medicine, Mayo Clinic, 200 First Street SW, Rochester, MN 55905 USA; 2 Present Address: Genomic Science and Precision Medicine Center, Clinical and Translational Sciences Institute, Medical College of Wisconsin, Milwaukee, WI 53226 USA

**Keywords:** Individualized medicine, Cancer treatment protocols, Transcriptome profiling, Pharmacokinetics, Genomic interpretation

## Abstract

**Background:**

The right drug to the right patient at the right time is one of the ideals of Individualized Medicine (IM) and remains one of the most compelling promises of the post-genomic age. The addition of genomic information is expected to increase the precision of an individual patient’s treatment, resulting in improved outcomes. While pilot studies have been encouraging, key aspects of interpreting tumor genomics information, such as somatic activation of drug transport or metabolism, have not been systematically evaluated.

**Methods:**

In this work, we developed a simple rule-based approach to classify the therapies administered to each patient from The Cancer Genome Atlas PanCancer dataset (*n* = 2858) as effective or ineffective. Our Therapy Efficacy model used each patient’s drug target and pharmacokinetic (PK) gene expression profile; the specific genes considered for each patient depended on the therapies they received. Patients who received predictably ineffective therapies were considered at high-risk of cancer-related mortality and those who did not receive ineffective therapies were considered at low-risk. The utility of our Therapy Efficacy model was assessed using per-cancer and pan-cancer differential survival.

**Results:**

Our simple rule-based Therapy Efficacy model classified 143 (5%) patients as high-risk. High-risk patients had age ranges comparable to low-risk patients of the same cancer type and tended to be later stage and higher grade (odds ratios of 1.6 and 1.4, respectively). A significant pan-cancer association was identified between predictions of our Therapy Efficacy model and poorer overall survival (hazard ratio, HR = 1.47, *p* = 6.3 × 10^− 3^). Individually, drug export (HR = 1.49, *p* = 4.70 × 10^− 3^) and drug metabolism (HR = 1.73, *p* = 9.30 × 10^− 5^) genes demonstrated significant survival associations. Survival associations for target gene expression are mechanism-dependent. Similar results were observed for event-free survival.

**Conclusions:**

While the resolution of clinical information within the dataset is not ideal, and modeling the relative contribution of each gene to the activity of each therapy remains a challenge, our approach demonstrates that somatic PK alterations should be integrated into the interpretation of somatic transcriptomic profiles as they likely have a significant impact on the survival of specific patients. We believe that this approach will aid the prospective design of personalized therapeutic strategies.

**Electronic supplementary material:**

The online version of this article (10.1186/s12885-018-4345-2) contains supplementary material, which is available to authorized users.

## Background

The right drug to the right patient at the right time is one of the promises of Precision or Individualized Medicine (IM) [1]. The integration of genetic information and targeted therapies into patient care is expected to increase the precision of individual patient’s treatment, resulting in improved outcomes [2–4]. Previous work has reported the successful use of genomics information to discover causal or driver mutations [5], guide treatment [6–9] in oncology, and investigated the feasibility of relating somatic alterations to known druggable targets [5, 10–15]. These studies mostly focused on drug target identification. However, there are additional factors that influence the fate of drug therapies. Pharmacokinetic (PK) genes mediate transport of drugs into and out of cells and their metabolic processing. For example, PK genes can be activated within tumor tissues and thereby compromise the efficacy of an administered therapy independent of the target. While interest in PK genes has recently increased for interpreting the effects of epigenetic and copy number alterations in recurrent ovarian cancer [16, 17], systematic pan-cancer investigation of the prevalence and impact of PK alterations using patient-level data has not been performed, despite being a well-understood mechanism.

The basic mechanisms of PK genes have been studied extensively [18], as have PK gene alterations’ impact on drug response [19–22]. It has been shown that specific mechanisms of PK activation are associated with resistant cancer cell lines and patient tissues [23–25]. Moreover, cancer cell lines may increase expression of drug metabolism genes active against administered therapies [26]. As a specific example for a commonly administered chemotherapy, the efficacy of 5-florouracil (5-FU) has been shown to be diminished by activation of the gene DPD, which degrades 5-FU [27], by altered expression levels of export genes [28], or by inhibited expression of metabolically activating enzymes [29], while greater efficacy is observed with genomic deletions of DPD [30]. Thus, the systematic integration of PK knowledge, which we defined as the known relationships between genes and therapies, into the interpretation of individual patient’s tumor genomics may improve IM in oncology.

In this study, we investigated the potential contribution of PK knowledge, specifically the individualized prediction of drug efficacy, to the interpretation of tumor gene expression from The Cancer Genome Atlas (TCGA). We focused on therapies administered early in each patient’s course of treatment, because TCGA data was derived from untreated primary tumors. We assessed patient data from multiple tumor types (pan-cancer). First, we developed a simple rule-based Therapy Efficacy model (TEM) using a small number of features that correspond to well-established PK mechanistic processes [31–33]. We used the TEM to evaluate the therapies administered to each patient, accounting for their corresponding transcriptomics data. Therapies were predicted to be effective or ineffective. Next, we used survival analysis to validate predictions made by our model. To perform survival analysis, we first defined two groups of patients. Patients who received a predicted ineffective therapy were considered to be at higher risk of cancer-related mortality, due to the lack of expected drug efficacy. Those who did not receive a predicted ineffective therapy were considered at lower risk of cancer-related mortality. We refer to these groups as high-risk and low-risk, respectively. We hypothesized that high-risk patients would experience poorer therapy efficacy and thereby shorter survival compared to low-risk patients. Therefore, we validated our TEM predictions using differential overall and event-free survival. Our TEM categorized 5% of the patients as high-risk who exhibit poorer survival compared to those who were low-risk. Our data indicated that the altered PK gene expression does not significantly impact the majority of cancer patients in this cohort. However, altered PK gene expression has significant impact on patients with these alterations and therefore could be taken into account when designing personalized treatment strategies.

## Methods

### Data and normalization

#### Tumor genomic and patient datasets

Data released during the Pan-Cancer initiative [34, 35] was downloaded from synapse.org. We used normalized gene expression from syn1695384. We refer to each cancer type by TCGA abbreviation. Patient’s clinical characteristics, follow-up, and treatment administration data were gathered from the public access portal (https://tcga-data.nci.nih.gov/tcga/dataAccessMatrix.htm). According to TCGA policies, genomic data were collected from pre-treatment tissues, limiting our knowledge of post-treatment genomics. No embargoed or limited access data was used. See Supplemental Text for further detail on how clinical covariates were coded. Patients with verified missing data (e.g. no administration times for the therapies administered) were excluded from our analysis.

#### Gene expression normalization

**Fig. 1:**
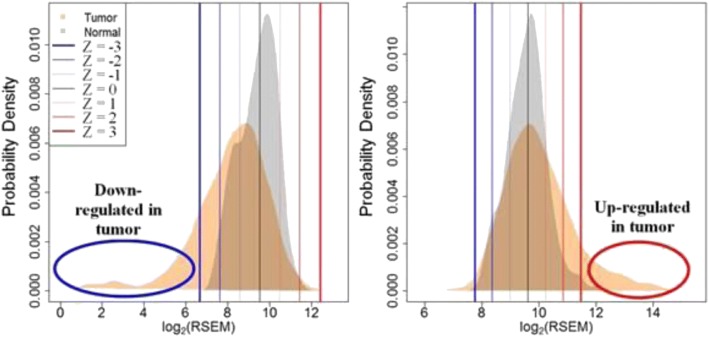
Human tumors may up- or down-regulate PK genes. Each gene was scored relative to a composite-normal reference to generate conservative estimates of aberrant somatic gene expression. RSEM normalized gene expression data were used. The expression score of each gene in each tumor sample is the signed Z-score relative to normal tissue samples. Example probability density distributions of gene expression for two genes are shown: (Left) drug importer SLC16A2 and (Right) drug exporter ABCC5

In order to quantify the level of gene expression in each tumor as high or low, we utilized a normal-tissue reference (Fig. 1). We considered the differential expression of an individual tumor sample compared to the expression level seen in a collection of normal-tissue samples. In order to achieve a more comprehensive (applicable to all cancer types) yet conservative estimate of aberrant gene expression, all normal-tissue samples within the dataset (*n* = 295) were combined to generate composite-tissue gene expression reference ranges. Tumor gene expression levels were transformed to their Z-score by median-centering each gene and normalizing by its median absolute difference [36], using the composite-tissue reference ranges. As a balance between the number of affected samples and effect size, unless otherwise stated, we used a threshold of Z ≥ 2 to define a “high” and Z ≤ − 2 a “low” gene expression level [37].

#### Gene-drug relationships

Gene-drug relationships for administered therapies were extracted from CPIC guidelines, PharmGKB pathways [38], and DrugBank [39, 40]. We reviewed the literature supporting each relationship in order to determine the genes most likely to be dominant for each effect.

#### Therapy normalization

Therapy administration information from TCGA could not be used without additional processing since drugs were referenced by generic name, brand name, or abbreviations. Therapy names were manually corrected for spelling mistakes and normalized to their US generic name. When a therapy refers to a combination of drugs, the therapy name was de-convoluted into a normalized set of therapy names. For instance, “Macdonald” was converted to fluorouracil, leucovorin and radiotherapy. Our manual review process leveraged many sources including: uptodate.com, chemocare.com, chemoregimen.com, dtp.nci.nih.gov, DGIdb [11], DrugBank [39], NCCN guidelines [41–44], RxNorm [45, 46], and the NCI Thesaurus. [47]

#### Defining eligible therapies for evaluation

Because TCGA samples are untreated, we focused on first-line therapies. NCCN guidelines for multiple cancer types were manually reviewed to identify common administration schedules for standard-of-care chemotherapies. After this review, we determined that any group of therapies initiated within the first 1.5 years after diagnosis was a reasonable pan-cancer approximation to first-line therapies, but will likely miss cases that experienced early disease progression. Of note, the majority of therapies administered after 1.5 years are after each patient’s event-free interval (defined below). Therefore, because our event-free survival analysis accounted for changes in disease status, it did not evaluate therapies administered after disease progression. Patients were considered low-risk until they received a predicted ineffective therapy, according to our model. When they did, their status was updated to high-risk.

It has been shown that early response to treatment can predict long-term outcomes [48–52]. Therefore, it is likely that the effects of early ineffective therapies will be evident, on average, in long-term outcomes data. Previous studies have demonstrated that tumor evolution is associated with chemotherapy resistance [53–56]. Our eligibility criteria have the added benefit of minimizing the potential effects of treatment-induced tumor evolution on our analysis. Determination of the most informative duration of pre-treatment genomics data for predicting therapy efficacy is beyond the scope of the current study.

### Deriving the therapy efficacy model

#### Defining tumor genomics features

**Fig. 2:**
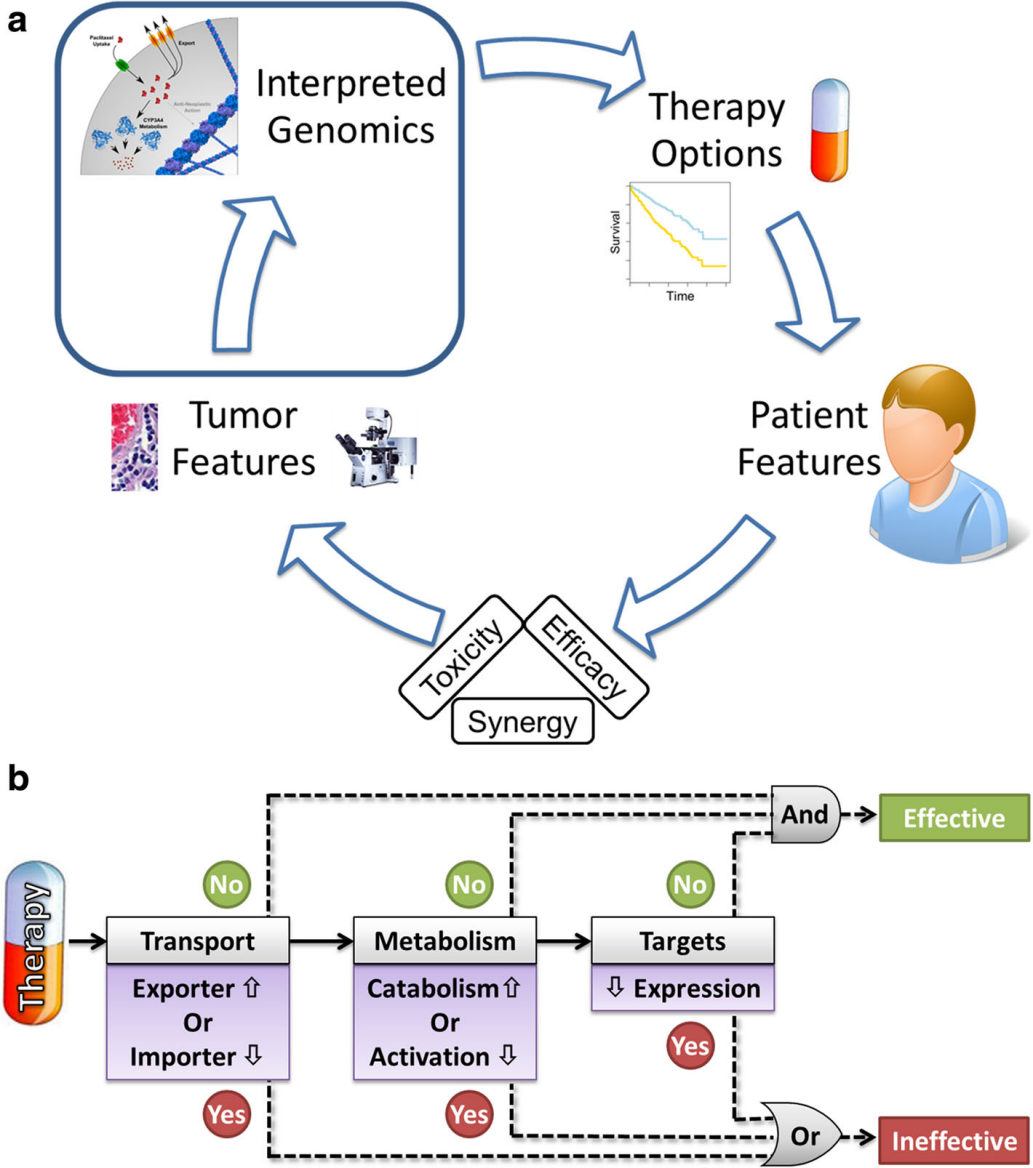
Clinical decision making is augmented by interpreted tumor genomics. **a** The clinical decision making cycle of therapy selection is multifaceted and can be informed by properly interpreted tumor genomics profiling. Developing high-confidence and mechanism-based algorithms for properly interpreting genomic information remains a clinical challenge. **b** We considered a set of rules based on tumor genomics features for interpreting somatic PK gene expression. From these rules, we developed a simple Therapy Efficacy model for interpreting if an administered therapy may be ineffective due to somatic PK gene expression

We developed our Therapy Efficacy model (TEM) using a simple rule-based approach that leveraged each patient’s tumor genomics features. Three features were considered, each hypothesized to negatively affect drug efficacy: one feature for drug transport, one for drug metabolism, and one for drug targets. Specifically: 1) low gene expression of drug import genes that bring the therapy into tumor cells or high expression of drug export genes that pump the therapy out of tumor cells (e.g. ABCC1 exports paclitaxel from tumor cells); 2) high expression of genes known to metabolically degrade the therapy, or, when pro-drug activation occurs within target cells, low expression of pro-drug activating genes (e.g. CYP3A4 breaks down paclitaxel within tumor cells); 3) low expression of target genes (Fig. 2; e.g. paclitaxel binds to tubulin proteins). We list additional and specific drug-gene examples that were important for our TEM, in our results.

#### Defining rules from tumor genomic features

Each tumor genomics feature corresponds to a different molecular mechanism (transport, metabolism, and target) and could be activated independently of the others, for a particular patient. Therefore, the assessment of each feature individually and their combinations is of interest. For individual-feature rules, we classified a therapy as ineffective if any gene within the patient’s tumor genomics profile fit the features’ criteria as described above. We considered three rules that utilized combinations of features. The first combination was an “Any-Hit” rule which was a simple Boolean combination of all individual-feature rules. The second combination was an “Any-PK-Hit” rule where a therapy is predicted to be ineffective if any gene met the criteria for either of the two PK features. Finally, we considered the features from statistically significant rules (see below for details) and combined them to generate our TEM. Our TEM is therefore derived from an empirical and iterative process from a small number of well-established features. Our TEM used two features: high expression of any exporter gene or high expression of at least two drug metabolism genes.

### Validating the therapy efficacy model using survival analysis

#### Classifying patients using rules from tumor genomic features

Patients who received a predicted ineffective therapy were considered high-risk, while patients who did not receive a predicted ineffective therapy were considered low-risk. We compared the overall and event-free survival of high-risk patients to low-risk patients to validate the predictions of tumor genomics rules, including our Therapy Efficacy model.

#### Survival analysis models

Using Cox regression, we first evaluated survival differences between high-risk and low-risk patients, per cancer. We computed univariate survival models that only used our patient classification, as well as multivariate survival models that adjusted for clinical covariates such as patient age and tumor stage. See Table 1 for the full list of covariates used for each cancer type. Statistical model results were summarized by hazard ratio (HR), the lower and upper bounds of the 95% confidence interval, and *p*-value. HRs greater than 1 indicated increased risk and values less than 1 indicated a protective association. We reported HRs of high-risk patients relative to low-risk patients. We used Kaplan-Meyer (KM) plots for visualization of differences between high- and low-risk patients.

**Table 1:** Number of samples available and covariates considered for each cancer type. Column names designate covariates with a bullet indicating inclusion of that covariate in survival models

	#T	#E	#N	TSS^a^	Age	Sex	Stage	Grade	LI^b^	Smoke status^c^	Tumor specific^d^
GBM	394	154	0	●	●	●					●
OV	573	262	0	●	●		●	●	●		●
BRCA	905	817	104	●	●		●				●
UCEC	473	333	5	●	●		●	●			
KIRC	505	470	65	●	●	●	●	●			
COAD	424	192	0	●	●	●	●		●		●
READ	168	71	0	●	●	●	●		●		●
BLCA	139	96	13	●	●	●	●			●	
LUAD	447	353	55	●	●	●				●	
LUSC	404	220	16	●	●	●				●	
HNSC	409	303	37	●	●	●	●	●		●	

#T, total number of tumor samples

#E, number of tumor

#N, number of tissue-matched normal samples

^a^Random intercept terms were included for Tissue Source Site

^b^Lymphatic Invasion; coded as “present” or not

^c^Cigarette smoking status

^d^Measures used in only one cancer type. GBM: Radiation dosage. OV: tumor residual disease. BRCA: menopause status, margin status. COAD and READ: history of colon polyps

Because we considered tumor genomic information, features may be transferrable across cancer types. However, due to many factors (including small numbers of cases for each cancer), observations of each rule’s HR may be variable. Therefore, we used random-effects meta-analysis to combine information across cancer types and derive a more robust estimate of the pan-cancer HR [57, 58]. In meta-analysis, it is assumed that each cancer type is an observation of the “true” pan-cancer HR. The number of high- and low-risk patients, the HR, and the confidence interval for the HR, from each cancer type were used in the meta-analysis. In addition to meta-analysis, we also computed a pan-cancer Cox regression, stratified by cancer type. Statistical models produced by meta-analysis and Cox regression were compared. Finally, we used 5-fold cross-validation repeated 50 times to assess variability in risk associated with each feature.

Overall survival times were calculated using the time to observed cancer-related mortality. Event-free survival was calculated using the time to any cancer-related event (cancer-related mortality, disease progression, recurrence, or beginning a new treatment) [59–62]. Subjects were censored when lost to follow-up. In all survival models, we required at least 5 patients to be in each group, at least 10 total observed events, and at least 20 total patients. Models were compared using likelihood ratio tests.

#### Software used

Analyses were conducted using the R programming language [59, 60, 63] (version 3.1.1), leveraging the survival (version 2.38.3), coxme (version 2.2.5), meta (version 4.3.0), and forestplot (version 1.1.0) packages.

## Results

### Classification of therapies and patients using rules from tumor genomic features

Our Therapy Efficacy model classified 229 therapies (9.0%) as ineffective within the context presented by the corresponding patient’s tumor genomics profile. These therapies were administered to 143 (5.5%) patients, leading to classification of these patients as high-risk. While high-risk patients were distributed across cancer types (Table 2), a higher proportion was observed in BRCA and OV. Differences in prevalence between cancer types also corresponded with moderate differences in clinical covariates; high-risk patients trended towards higher grade, later stage, and younger age at cancer diagnosis. Further details on the cohort characteristics, number of genes annotated for each therapy, and the fraction of administered therapies affected by PK gene expression, are available in Additional file 1: Figures S1, S2.

**Table 2:** Per-cancer characteristics of low- and high-risk patients

	Low-risk cohort						High-risk cohort					
	N	Age	Stage 1 or 2	Stage 3 or 4	Grade 1 or 2	Grade 3 or 4	N	Age	Stage 1 or 2	Stage 3 or 4	Grade 1 or 2	Grade 3 or 4
Any-Hit Model												
GBM	125	62 ± 13.3	–	–	–	–	9	52 ± 23.7	–	–	–	–
BRCA	697	59 ± 13.3	533	164	–	–	118	53 ± 13.3	96	22	–	–
OV	159	58 ± 10.4	9	150	17	142	103	57 ± 13.3	10	93	19	84
KIRC	456	61 ± 13.3	389	67	211	245	14	58.5 ± 11.1	2	12	1	13
LUAD	319	67 ± 10.4	246	73	–	–	10	62.5 ± 12.6	6	4	–	–
UCEC	289	63 ± 10.4	226	63	164	125	44	61.5 ± 9.6	13	31	12	32
HNSC	287	61 ± 11.9	136	151	199	78	16	56.5 ± 10.4	14	2	11	5
LUSC	198	68.5 ± 8.2	152	46	–	–	14	65.5 ± 8.9	12	2	–	–
Therapy Efficacy Model												
GBM	127	61 ± 13.3	–	–	–	–	7	52 ± 23.7	–	–	–	–
BRCA	776	58 ± 13.3	602	174	0	0	39	53 ± 13.3	27	12	–	–
OV	228	58 ± 11.1	16	212	31	197	34	60.5 ± 11.9	3	31	5	29
KIRC	463	61 ± 13.3	390	73	212	251	7	54 ± 8.9	1	6	0	7
LUAD	321	67 ± 10.4	247	74	0	0	8	67 ± 10.4	5	3	–	–
UCEC	310	63 ± 10.4	231	79	168	142	23	61 ± 10.4	8	15	8	15
HNSC	288	61 ± 11.9	137	151	200	78	15	57 ± 13.3	13	2	10	5
LUSC	202	68.5 ± 8.2	155	47	0	0	10	65 ± 8.9	9	1	–	–

### Per-Cancer survival analysis

We utilized survival analysis comparing high- and low-risk patients to validate the predictions of our simple TEM. Survival analysis was first applied to each cancer type independently. Differential survival was assessed using the TEM classification (univariate) and with adjustment for clinical covariates (multivariate; see Table 3). For univariate analysis, only KIRC showed a statistically significant association. However, the majority of cancer types exhibited survival patterns consistent with poorer survival for high-risk patients. In multivariate analysis, four out of eight cancer types demonstrated statistically significant poorer survival for high-risk patients: GBM, BRCA, KIRC, and HNSC. Comparing univariate and multivariate models, GBM was the only cancer type for which the risk associated with the TEM significantly differed, but there were substantial clinical differences (e.g. age and radiation). While each per-cancer model identified a small number of high-risk patients, a significant association with survival was observed in multiple cancers.

**Table 3:** Per-cancer Therapy Efficacy model performance assessed using overall survival

	Univariate^b^		Multivariate^c^	
	HR [95% CI]^a^	*p*-value	HR [95% CI]	*p*-value
GBM	0.87 [0.38, 2.01]	7.5 × 10^− 1^	1.84 [1.17, 2.89]	7.9 × 10^− 3^
BRCA	1.96 [0.75, 5.12]	1.7 × 10^−1^	1.80 [1.40, 2.30]	3.0 × 10^−6^
OV	1.56 [0.94, 2.57]	8.4 × 10^−2^	1.46 [0.88, 2.42]	1.4 × 10^− 1^
KIRC	7.75 [3.25, 18.50]	3.9 × 10^− 6^	3.07 [2.18, 4.33]	1.7 × 10^− 10^
LUAD	1.53 [0.47, 4.94]	4.8 × 10^−1^	1.65 [0.94, 2.91]	8.0 × 10^− 2^
UCEC	1.60 [0.48, 5.32]	4.4 × 10^−1^	0.94 [0.58, 1.54]	8.1 × 10^− 1^
HNSC	1.39 [0.63, 3.08]	4.2 × 10^−1^	1.59 [1.05, 2.42]	2.9 × 10^−2^
LUSC	0.67 [0.21, 2.14]	4.9 × 10^−1^	0.63 [0.35, 1.12]	1.2 × 10^−1^

^a^The hazard ratio (HR) and the bounds of its 95% confidence interval (CI)

^b^The HR associated with being in the high-risk cohort

^c^The HR associated with being in the high-risk cohort, after accounting for clinical covariates as itemized in Table 1

**Fig. 3:**
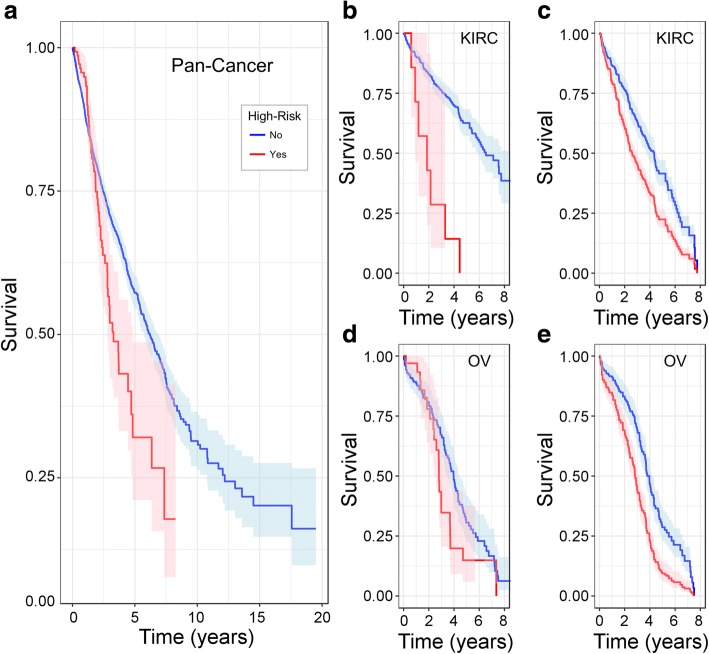
Our Therapy Efficacy model identified high-risk patients who received therapies with specific PK mechanisms activated within their tumors. **a** We first plot the Kaplan-Meyer survival curves of the pan-cancer cohort classified by our genomics-based efficacy model based on high expression of the exporters or metabolizers of the drugs administered. Shaded bands indicate 95% confidence intervals. Analogous per-cancer survival curves are shown for (**b**) KIRC and (**d**) OV. In this retrospective study, cohorts were not balanced with respect to disease state or clinical characteristics (Additional file 1: Figure S1). Thus, we plot survival curves adjusted to a uniform cohort of 50 year olds with stage-3 grade-3 (**c**) KIRC or (**e**) OV. Analogous plots are shown for all cancer types in Additional file 1: Figure S3. We assessed statistical significance using Cox regression with results shown in Table 3 and Figs. 4, 5 and 6

Kaplan-Meyer (KM) plots were used to visualize differential survival between high- and low-risk patient cohorts (Fig. 3 and Additional file 1: Figure S3). The high- and low-risk cohorts were not balanced with respect to clinical characteristics. KM plots do not provide sufficient resolution to account for the differences in survival due to different covariate balance. Therefore, we calculated unadjusted and adjusted KM survival curves to more clearly represent the survival associations for the TEM.

### Pan-Cancer survival analysis

**Fig. 4:**
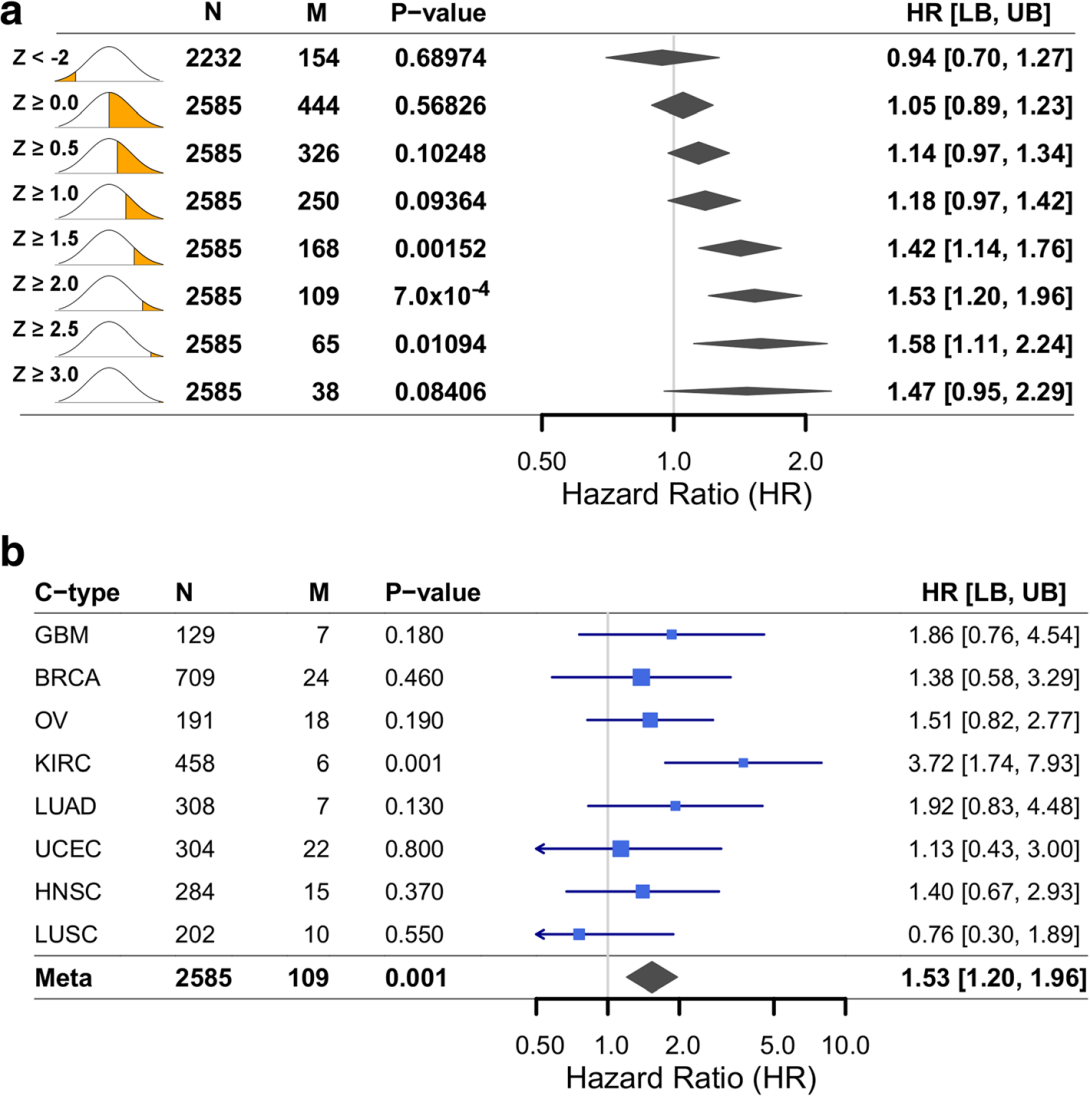
High expression of drug export genes for administered therapies is associated with poorer overall survival. **a** Our pan-cancer analysis revealed a statistically significant association between high expression levels of genes known to export therapies administered to each patient and overall survival. Pan-cancer cohort size is indicated (N) along with the number of patients affected (M) Varying the expression threshold used identified a tradeoff between the number of patients affected and HR magnitude. A distribution diagram highlights the analogous region considered for each model. Models are summarized by *p*-value, HR, and bounds of the 95% confidence interval. Meta-analyses are summarized by a diamond centered on the HR and its width extending to the confidence interval bounds. **b** For the Z ≥ 2 criteria, per-cancer models are summarized in a Forest plot. For each cancer type, the HR is marked and scaled by M; a line extends to the confidence interval bounds

Across all patients, we first tested single-feature rules (Table 4 and Additional file 1: Figure S4). Drug export (HR = 1.49 [1.13, 1.96], *p* = 4.7 × 10^− 3^, Fig. 4 and Additional file 1: Figure S5) and metabolism (HR = 1.73 [1.31, 2.28], *p* = 9.3 × 10^− 5^, Additional file 1: Figures. S6 and S7) both demonstrated statistically significant associations between high-risk patients and poorer survival. Neither therapy import nor target gene expression demonstrated significant survival associations.

**Table 4:** Pan-cancer Therapy Efficacy model performance assessed using overall survival

					Univariate		Multivariate	
Rule^a^	Genomics features	Low-risk (N)	High-risk (N)	High-risk (%)	HR [95% CI]	*p*-value	HR [95% CI]	*p*-value
Export	1	2749	109	3.8	1.49 [1.09, 2.05]	1.2 × 10^−2^	1.49 [1.13, 1.96]	4.7 × 10^− 3^
Import	1	2777	81	2.8	0.90 [0.61, 1.33]	6.0 × 10^−1^	1.00 [0.76, 1.32]	1.0 × 10^0^
Metabolism	1	2642	216	7.6	1.26 [0.94, 1.69]	1.3 × 10^−1^	1.12 [0.85, 1.48]	4.2 × 10^− 1^
Metabolism^b^	1	2809	49	1.7	1.74 [1.09, 2.77]	2.1 × 10^− 2^	1.73 [1.31, 2.28]	9.3 × 10^− 5^
Target	1	2780	78	2.7	1.12 [0.76, 1.66]	5.6 × 10^− 1^	1.07 [0.81, 1.41]	6.3 × 10^− 1^
Any-Hit	3	2530	328	11.5	1.21 [0.94, 1.55]	1.4 × 10^− 1^	1.17 [0.88, 1.53]	2.8 × 10^− 1^
Any-PK-Hit	2	2660	198	6.9	1.26 [0.97, 1.64]	7.8 × 10^−2^	1.27 [0.96, 1.67]	9.4 × 10^− 2^
Efficacy Model	2	2715	143	5.0	1.41 [1.06, 1.88]	1.9 × 10^−2^	1.47 [1.11, 1.93]	6.3 × 10^− 3^

^a^Rules based on tumor-genomic features; see Methods for definitions

^b^Requiring at least two metabolism genes affected; see Additional file 1: Figure S1 for additional comparisons

We next constructed rules from combinations of features and tested them using survival models (Fig. 3 and Table 4). The Any-Hit model was consistent with poorer survival, but was not statistically significant. Both the Any-PK-Hit and TEM demonstrated statistically significant associations between high-risk patients and poorer survival (HR = 1.27 [0.96, 1.67], *p* = 9.4 × 10^− 2^ and 1.47 [1.11, 1.93] 6.3 × 10^− 3^, respectively). Associations were consistent between univariate and multivariate models. KM survival curves for the high-risk and low-risk cohorts were used to visualize these associations and are presented in Fig. 3, Additional file 1: Figures S1 and S3.

We tested if inclusion of scores from the TEM was a significant improvement. Details are presented in our Supplemental Information. Briefly, inclusion of the TEM classification was significant by the likelihood ratio test (*Χ*^2^(1) = 5.4, *p* = 0.02) over a multivariate model including all covariates.

### Analysis of tumor genomics features

We analyzed the prevalence of each genomics feature across the cohort and investigated which specific drug-gene relationships were most commonly affected by altered PK gene expression levels for the PK genes relevant to each patient’s treatment.

#### Therapy export

Across cancer types, 4.2% (*n* = 109) of patients were affected by high exporter gene expression for a therapy they received. As described above, this feature was associated with increased cancer mortality risk (Table 4 and Fig. 4; HR = 1.49 [1.13, 1.96], *p* = 4.7 × 10^− 3^) and event-free survival (HR = 1.43 [1.11, 1.85], *p* = 5.9 × 10^− 3^; Additional file 1: Figure S5). Lowering the threshold used to classify gene expression as “high” lead to more patients identified at a lower HR and increasing the threshold lead to a similarly high HR. This indicated that different expression levels of drug export genes may indicate different levels of impact on drug efficacy.

The poorer survival associated with high expression of drug export genes was influenced by many individual drug-gene relationships. The most recurrent alteration observed was elevation of ABCC1 expression in 48 patients receiving doxorubicin and/or paclitaxel from across six cancer types. The next most recurrent alterations were observed for three further independent groups of patients exhibited high expression of ABCC2 (17 patients), ABCG2 (12 patients), and ABCC10 (8 patients). While these patients have high expression of one of these four genes, their treatment regimen may differ, impacting different therapies as ineffective across patients. Our model is able to integrate across these multiple drug-gene relationships. Specific patient cases are highlighted in Table 5. Thus, increased exporter gene expression is a pan-cancer feature affecting a modest fraction of cancer patients, but which may convey a significant impact on drug efficacy for individual patients.

**Table 5:** Selected cases of aberrant PK or target expression likely affecting administered therapies

Ctype	Patient	Primary regimen	Additional therapy	Age	Stage	Grade	Details^a^	Target ^c^	Metabolism	Transport
BRCA	TCGA-E2-A1LB	Cyclo-phosphamide Paclitaxel Doxorubicin Anti-Hormone^b^	Trastuzumab	41	2	–	ER-		AKR1A1(3.01) - Dox	ABCC2(4.77) - Pac
BRCA	TCGA-E2-A158		–	43	2	–	ER-	MAP2(−3.56) - Pac MAP4(− 3.29) - Pac		ABCC1(2.27) - Dox
BRCA	TCGA-E2-A14V		Lapatinib	53	2	–	ER+	EGFR(−4.65) - Lap	CYP2C8(2.2) - Pac	
BRCA	TCGA-AO-A0J3		Fluorouracil Methotrexate	67	2	–	ER+	MAP4(−3.11) - Pac		ABCC5(2.21) - 5FU,Meth FOLR1(− 3.42) - Meth
BRCA	TCGA-BH-A0BA		–	51	3	–	ER+		NQO1(3.22) - Dox	ABCC2(3.09) - Pac
BRCA	TCGA-AO-A0JE		Trastuzumab	53	3	–	ER-Margin+			ABCC1(2.73) - Pac, Dox ABCC2(3.86) - Pac, Dox
OV	TCGA-09-0367	Carboplatin Doc/Paclitaxel	Gemcitabine	67	3	3	LI + TRD+	MAP2(−3.66) - Pac MAP4(−3.96) - Pac	NT5C(3.78) - Gem	SLC31A1(−2.67) - Car
OV	TCGA-23-1123		Doxorubicin	59	3	3	LI + TRD+		NR1I2(3.42) - Pac	RALBP1(4.17) - Car,Dox
OV	TCGA-24-1558		Doxorubicin	73	3	3	TRD+	MAP2(−4.71) - Pac,Doc		ABCC1(6.49) - Gem,Dox AABCC10(2.66) - Gem,Dox
OV	TCGA-30-1860		Doxorubicin	58	3	3	TRD+	TOP2B(−4.39) - Pac,Dox MAP2(− 5.04) - Pac,Dox		
OV	TCGA-61-1724		Doxorubicin Gemcitabine	47	3	3	LI+	MAP2(−4.57) - Doc	DCTD(4.46) - Gem	
OV	TCGA-61-1741		Tamoxifen	76	3	3	TRD+		CYP2C8(3.19) - Pac,Tam NR1I2(3.94) - Pac,Tam	SLC31A1(−3.21) - Car
KIRC	TCGA-CJ-4638	Gemcitabine Fluorouracil	Bevacizumab	46	4	4	–		NT5C(3.84) UPB1(3.87) DPYS(2.33) - Gem,5FU	
LUAD	TCGA-50-5072	Doc/Paclitaxel Carbo/Cisplatin		74	3	–	reformed smoker			ABCC2(5.12) - Doc,Pac
UCEC	TCGA-AP-A052	Carboplatin Paclitaxel	Gemcitabine	59	4	3		CMPK1(−4.12) - Gem	NR1I2(3.92) - Pac	
UCEC	TCGA-AP-A05D			67	3	3		MAPT(−3.05) - Pac TUBB1 (−2.43) - Pac		SLC31A1(− 3.7) - Carb
UCEC	TCGA-AP-A0LI			67	3	3		MAP2(−5.57) - Pac MAPT(−2.21) - Pac		SLC31A1(−4.11) - Carb
UCEC	TCGA-AX-A0IS		Gemcitabine Brivanib	52	1	2		MAP2(−3.09) - Pac MAP4(− 3.27) - Pac	DCTD(3.49) - Gem	
UCEC	TCGA-AX-A1CR			70	2	3			NR1I2(3.18) - Pac	SLC31A1(−5.04) - Carb
UCEC	TCGA-BS-A0TE		Doxorubicin Topotecan	35	4	3			AKR1C3(4.88) NQO1(3.68) NR2I1 (2.76) - Dox,Pac	ABCC2(5.22) - Top
HNSC	TCGA-CR-7404	Carboplatin Paclitaxel	Cepecitabine Cetuximab	53	1	–	never smoker	MAP2(−3.86) - Pac		ABCC5(5.08) - Cap
HNSC	TCGA-DQ-7589		Docetaxel Fluorouracil	70	1	–	reformed smoker			ABCC1(5.83) - Doc,5FU,Pac ABCC5(4,74) - Coc,5FU,Pac
LUSC	TCGA-46-6026	Doc/Pacletaxel	Gemcitabine Paraplatin	81	2	–	reformed smoker	CMPK1(−3.05) - Gem	CYP2C8(3.02) - Pac NR1I2(3.86) - Pac	
LUSC	TCGA-94-7033		Cisplatin	73	1	–	reformed smoker	MAP4(−4.71) - Doc		SLC31A1(−3.26) - Cis

^a^Specific details for each case: ER, estrogen receptor; Margin, presence of tumor cells in surgical margins; TRD, rumor residual disease; LI, lymphatic invasion

^b^Anti-hormone therapies; three received anastrazole, one tamoxifen

^c^When multiple targets are lowly expressed, representatives are shown for brevity

Target or PK genes of drugs for the administered regimen that exhibit differences from normal-tissue expression levels are shown with their Z-scores. After the Z-score, we indicate which administered therapy is directly affected by the expression change using abbreviated names

#### Therapy metabolism

Across cancer types, 7.6% (*n* = 216) of patients were affected by increased gene expression of any drug metabolism gene active against the therapies they received, while 1.7% (*n* = 49) of patients were affected by concurrent high gene expression of at least two drug metabolism genes. Activation of any drug metabolism gene was consistent with poorer overall (HR = 1.27 [0.94, 1.71], *p* = 0.12, Additional file 1: Figure S6) and event-free survival (HR = 1.32 [0.93, 1.88], *p* = 0.11, Additional file 1: Figure S7). However, high expression of at least two drug metabolism genes was associated with poorer overall survival (HR = 1.73 [1.31, 2.28], *p* = 9.3 × 10^− 5^, Table 4). Varying the threshold for calling gene expression “high,” lead to an increased HR. This suggests that different expression levels of drug metabolism genes may indicate different levels of impact on drug efficacy.

Many individual relationships contributed to the association between administered therapies and the drug metabolism genes that degrade them within cancer cells. For example, we identified 41 patients in two cancer types receiving tamoxifen where the genes UGT1A10, UGT2B15, or CYP2D6 were highly expressed. These genes are well established components of tamoxifen’s metabolic pathway [64]. Across 5 cancer types, 109 patients who received paclitaxel exhibited activation of NR1I2, many with concurrent activation of CYP2C8 or CYP3A4 – known members of paclitaxel’s metabolic pathway [65]. Further relationships are identified including high expression of the gene NQO1 in 33 patients receiving doxorubicin and the gene NT5C in 17 patients receiving gemcitabine. These and other relationships between administered therapies and the genes that metabolize them within tumor cells contribute to the survival association implicated by our pan-cancer survival model.

#### Therapy targets

**Fig. 5:**
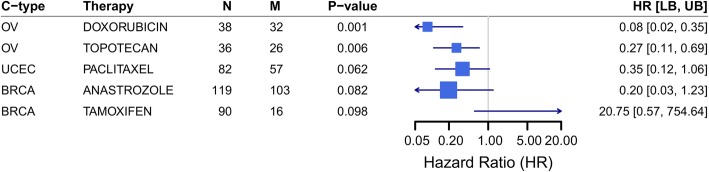
Low expression of drug target genes for administered therapies is associated with patient survival in a mechanism-dependent manner. For specific therapies and per-cancer, examples with at least five patients in each group are shown. Data are presented as in previous figures; N, the number of patients administered the therapy; M, the number of patients with low levels of at least one of the therapy’s targets

Across cancer types, 241 (8.4%) of patients were affected by low gene expression of any drug target gene for their administered chemotherapy, while 2.7% (*n* = 78) of patients were affected by concurrently low gene expression of at least two drug targets. We tested the association between survival and low drug target gene expression for administered therapies, but found no overall statistical association, either for one gene (HR = 0.99, *p* = 0.92) or two genes (HR = 1.12, *p* = 0.56). However, specific therapies did exhibit statistically significant associations with overall survival. The direction of association was mechanism-dependent (Fig. 5). Among the most frequent drug target associations were with MAPT, MAP2, and MAP4 for patients receiving paclitaxel or docetaxel (Table 5). In total, 110 patients across 6 cancer types were affected by this mechanism. These three MAP proteins associate with microtubules and function to stabilize them in a phosphorylation-dependent manner [65, 66]. Increased MAP4 expression has been previously shown to enhance sensitivity to paclitaxel [67]. Thus, our analysis has recapitulated this known mechanism and demonstrated its pan-cancer prevalence. Another frequent alteration was low expression of growth factor receptors for patients receiving therapies that target them. For example, eight patients across four cancer types were identified with low expression of EGFR when receiving cetuximab or lapatinib, or of ERBB2 when receiving erlotinib. Additional individual patient examples included 14 patients receiving kinase inhibitors for which the primary target (KIT, RET, MTOR) was lowly expressed and 12 patients receiving tamoxifen when ESR2 was lowly expressed. These cases demonstrate the potential for individualized tumor genomics to inform the expected efficacy of administered therapies.

Platinum-based therapies are cytotoxic agents that nonspecifically target cellular DNA and their efficacy has been previously associated with the expression level of multiple genes including HMGB1 [68, 69], a critical chromatin modifier. HMGB1 has been shown to recognize and bind to platinum-induced damage [70, 71] and affect repair [72]. We investigated the association of HMGB1 expression levels in OV and identified a negative association with overall survival (Additional file 1: Figure S8; HR = 2.07 [1.13, 3.79]; *p* = 1.6 × 10^− 2^). Thus, our analysis supports the mechanism of HMGB1 counteracting the action of platinum therapies.

#### Cross-validation

We used repeated 5-fold cross-validation to assess variability in outcome associations. Therapy export was included in all cross-validated models with a similar effect size to our overall model (median HR = 1.47 ± 0.12). Therapy metabolism was included in 64% of models and both it and our TEM showed consistent effect sizes (median HR = 1.70 ± 0.13 and 1.52 ± 0.11, respectively). These results suggest that combinations of PK features, expected a priori to complement one another [31–33], validate from a data-driven perspective and our model is robust to variation in cohort.

#### Interactions between concurrently administered therapies

**Fig. 6:**
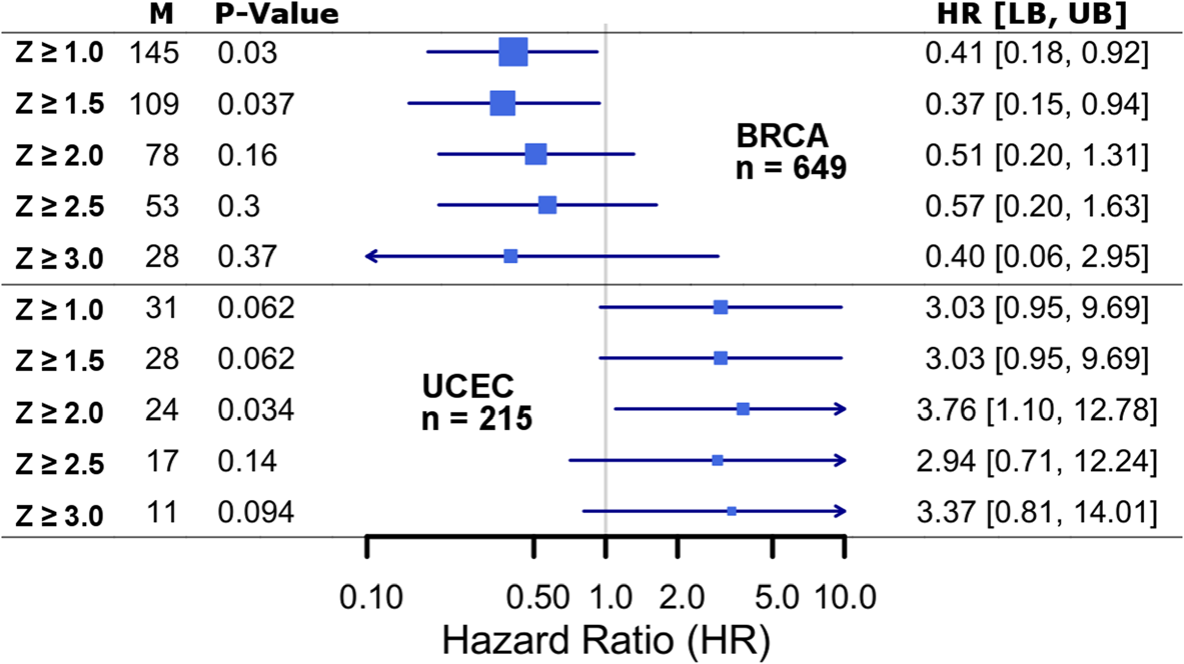
Genes involved in drug metabolism of one therapy may be the target of another therapy. High drug metabolism gene expression in BRCA was protective. This is the opposite association as expected and observed in other cancer types. The upper panel shows the survival association with high expression levels of drug metabolizing genes in BRCA, across a series of threshold values. Analogous data for UCEC is shown below, for comparison. We believe this association can be explained by interactions between the molecular mechanisms of cytotoxic therapies and anti-hormone therapies; see Discussion

In our analysis, the association between expression of metabolic genes and survival in BRCA (Fig. 6) was the opposite as expected and for which was observed in other cancer types. For this reason, BRCA was excluded from the analysis of therapy metabolism presented above. We believe that this observation can be interpreted by considering the other concurrently administered therapies (in the same regimen). In BRCA, patients are administered a combination of anti-hormone therapy, typically CYP19A1 (aromatase) inhibitors, and cytotoxic chemotherapy, usually taxanes. Taxanes such as paclitaxel are metabolized by CYP19A1 and this relationship is representative of the majority of drug metabolism cases identified in BRCA. We believe the protective effect associated with drug metabolism in BRCA is conferred by anti-hormone therapy activity, rather than metabolic activity of CYP19A1 on taxanes. Analogous trends can be seen for CYP3A4. Specific roles for CYP3A4 and CYP19A1 in breast cancer therapy response have been reviewed [73, 74], but only preliminary considerations have been made for modeling the concurrent and opposing activities of these enzymes on therapies within the same treatment regimen. These types of interrelationships between multiple therapies and genes will require further development so that genomic effects on concurrently administered drugs may be properly interpreted.

### Individual patient cases exhibiting multiple alterations

The associations between PK gene expression features and survival, presented above, defined a cohort of high-risk patients. These patients’ pre-treatment tumors exhibited features likely to indicate intrinsic resistance to the therapies they received. We summarize in Table 5, selected cases per cancer type where we believe prospective PK considerations during the interpretation of tumor genomics could have been of clinical benefit. Cases were selected to represent a diversity of clinical presentations. For example, we identified gene expression changes that indicated poor chemotherapy efficacy in 6 BRCA patients. These example patients had different hormone receptor status and therefore received differing anti-hormone therapies. However, we identified transcriptomic alterations that potentially affected the chemotherapies administered to each of these patients. The full list of cases is available in Additional file 2: Table S1. These observed alterations require additional investigation. However, we believe they demonstrate that PK considerations will be a critical component of interpreting tumor genomics for IM in oncology.

## Discussion

In this study, we have demonstrated that tumor genomics can be used to inform a deeper understanding of therapy efficacy. We used patient-level transcriptomic profiles to predict if administered therapies were rendered ineffective by somatic PK gene expression. Our Therapy Efficacy model (TEM) could be used to help select drugs that are the most appropriate for a patient, or used to identify patients that are less likely to respond to a therapy. In this study, we assumed that patients who are less likely to respond to their therapies would be at higher risk of cancer-related mortality. Therefore, we used survival analysis to validate predictions made by our TEM. While the level of detail in clinical annotations and follow-up time in TCGA data are not ideal, we believe they are sufficient to identify trends in overall and event-free survival as indirect measurements of therapy efficacy. The genomics data used was derived from pre-treatment tissue. It is expected that the occurrence of PK gene expression alterations will be higher for post-treatment samples due to therapy-induced selection. Scenarios where these assumptions do not apply represent limitations to our current study. While we did not detect altered somatic PK gene expression for the majority of patients, when present, it was significantly associated with poorer survival. We therefore believe that the features leveraged by our TEM will be important for interpreting tumor genomics profiles for individual patients.

The TEM is based on a small set of well-established PK features. They were individually tested using cross-validation which showed consistency in outcome associations. As the number of patients affected by each rule is relatively small, each fold may have too few cases to generate a robust estimate. Inclusion of the PK features we have considered in cross-validated models at comparable effect sizes to our overall model indicates a robust association for these rare events. As cancer genomics datasets increase, the number of cases with altered somatic PK gene expression is also likely to increase. Additionally, recurrent disease tends to be more resistant to baseline therapies, making adaptation through altered PK genes more likely. Therefore, assessing therapy efficacy using PK features such as we have considered, will be increasingly important.

The strongest PK features by survival analysis were high expression of genes that export or metabolize administered therapies. Influx genes were considered, but no significant association with survival was identified. We believe the difference in cancer mortality risk (quantified by HRs) between therapy importers and exporters can be simply explained by the inherent nature of the two systems; activation of a single exporter may convey sufficient efflux to impair the efficacy of a therapy, while inactivation of all importer genes may be required to sufficiently block influx of a therapy. Associations with target-gene expression depended on each target’s mechanism-of-action. Importantly, the risk estimates we have identified are comparable in incidence and magnitude to well-established germline mutations in cancer susceptibility genes [75] and to those observed for canonical cancer driver mutations [34]. Thus, we have established that specific PK mechanisms may be altered within human tumors and that a subset of them may lead to decreased therapy efficacy, independent of the drug target. Additionally, these features can be identified in pre-treatment tumor data and could be used to generate individualized predictions of therapy efficacy.

In predicting if a patient was high- or low-risk using our TEM, we assumed that receiving any ineffective therapy would negatively affect long-term survival outcomes. Treatment regimen are diverse [76–78] and have often been developed by balancing efficacy and toxicity, and relying on different individual therapies that have synergistic or complementary effects. Accounting for the relative contribution of each therapy within a regimen is left for future study. Further, the relative impact of altered PK gene expression on each therapy within a regimen could differ and may depend on each therapy’s mechanism-of-action. For example, their impact on cytotoxic agents may differ from molecularly targeted agents or hormone therapies. Additionally, contributions to PK activity from epigenetic regulation, somatic and germline mutation, and copy number variation may be integrated with gene expression levels to form a more comprehensive model of therapy efficacy. That is, we have considered the downstream readout of genomic regulation in pre-treatment samples: gene expression. However, cancer cells’ ability to resist chemotherapies may depend upon their ability to change their gene expression after treatment initiation. Their ability to change gene expression will depend on the epigenetic state of the drug’s corresponding PK genes. Additionally, somatic activating and inactivating mutations will modulate the expected positive correlation between gene expression and gene activity. Therefore, we are in the process of building a more advanced TEM using machine learning techniques that can integrate data types and weigh the relative contribution of each gene, leading to greater resolution and a more detailed interpretation of each patient’s tumor genomics data.

## Conclusions

In this study, we have established a simple rule-based Therapy Efficacy model for interpreting if a therapy will be ineffective for a specific patient by accounting for the patient’s somatic PK transcriptomic data. We validated the utility of these predictions by demonstrating that patients who were administered predicted ineffective therapies exhibited poorer survival than patients who were not administered predicted ineffective therapies, across cancer types. Thus, we suggest that PK-based guidelines could be integrated into clinical decision making to more effectively evaluate treatment regimen for individual cancer patients. These analyses demonstrate that somatic PK activity is likely to be important for IM in oncology.

## Additional files

Additional file 1: PK gene profiles across human cancers. This file contains additional text and figures that support the analyses presented in the main text. (DOCX 1543 kb)

Additional file 2: **Table S1.** This data file contains the full list of TCGA cases for which the TEM identified a potentially relevant interaction between PK gene expression levels and at least one administered therapy. (XLSX 41 kb)

## Abbreviations

CPIC: Clinical Pharmacogenetics Implementation Consortium
EFS: Event-free Survival
HR: Hazard Ratio
IM: Individualized Medicine
NCCN: National Comprehensive Cancer Network
OS: Overall Survival
PK: Pharmacokinetic
TCGA: The Cancer Genome Atlas
TEM: Therapy Efficacy Model
