# Correction to: the impact of pharmacokinetic gene profiles across human cancers

**DOI:** 10.1186/s12885-018-4593-1

**Published:** 2018-07-18

**Authors:** Michael T. Zimmermann, Terry M. Therneau, Jean-Pierre A. Kocher

**Affiliations:** 1 Division of Biomedical Statistics and Informatics, Department of Health Sciences Research, College of Medicine, Mayo Clinic, 200 First Street SW, Rochester, MN 55905 USA; 2 Present Address: Genomic Science and Precision Medicine Center, Clinical and Translational Sciences Institute, Medical College of Wisconsin, Milwaukee, WI 53226 USA

## Correction to: BMC Cancer (2018) 18:577 DOI https://doi.org/10.1186/s12885-018-4345-2

It has been highlighted that in the original manuscript [1] Fig. 1 was omitted and Fig. 2 appeared twice. This Correction article shows the correct Fig. 1. The original article has been updated.

**Fig. 1:**
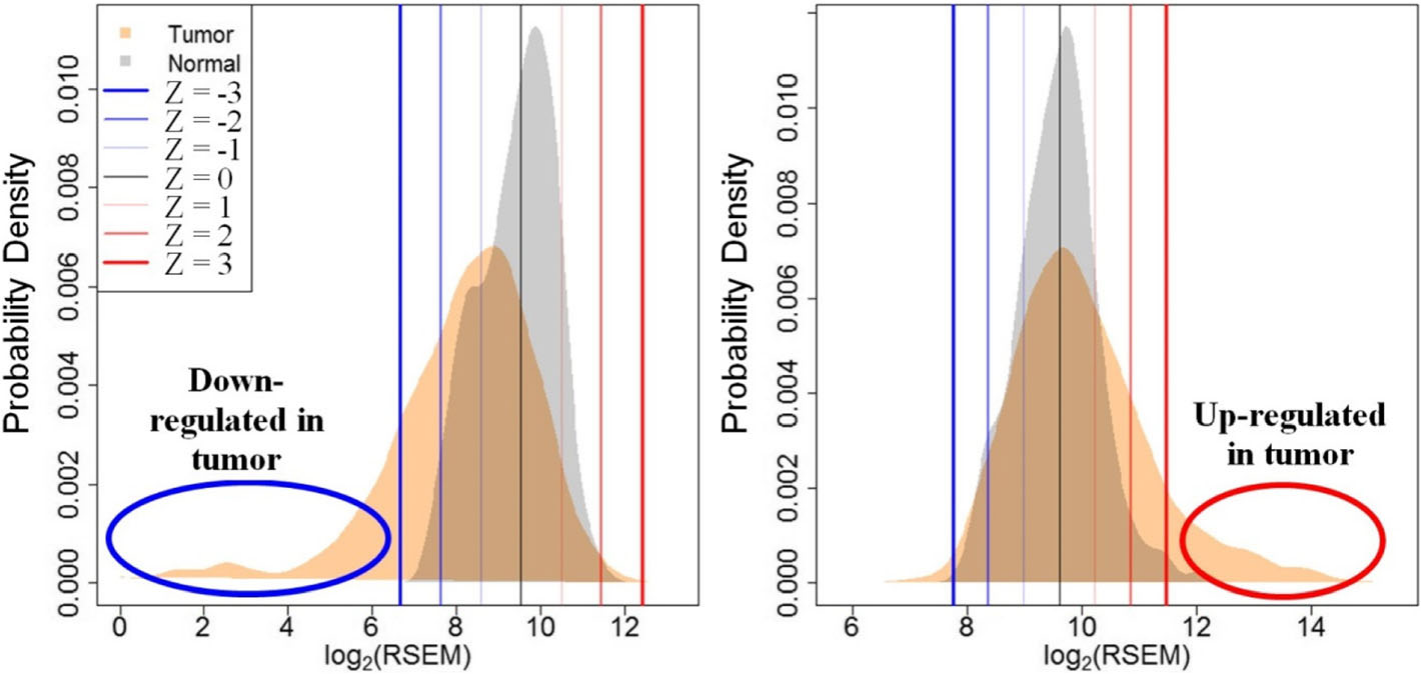
Human tumors may up- or down-regulate PK genes. Each gene was scored relative to a composite-normal reference to generate conservative. Q6 f1:2 estimates of aberrant somatic gene expression. RSEM normalized gene expression data were used. The expression score of each gene in each tumor. f1:3 sample is the signed Z-score relative to normal tissue samples. Example probability density distributions of gene expression for two genes are shown: f1:4 (Left) drug importer SLC16A2 and (Right) drug exporter ABCC5
