# Supplementary material for: The impact of pharmacokinetic gene profiles across human cancers
Source: BMC Cancer. 2018 May 21;18:577. doi: 10.1186/s12885-018-4345-2 (PMC5963084; doi:10.1186/s12885-018-4345-2)
Supplement: Supplementary file 1 — PK gene profiles across human cancers. This file contains additional text and figures that support the analyses presented in the main text. (DOCX 1543 kb) [file 12885_2018_4345_MOESM1_ESM.docx]

# Supplemental Informaiton to: “The Impact of Pharmacokinetic Gene Profiles across Human Cancers”

Michael T. Zimmermann^1^, Terry M. Therneau^1^, Jean-Pierre Kocher^1,*^

^1^Division of Biomedical Statistics and Informatics, Department of Health Sciences Research, College of Medicine, Mayo Clinic, 200 First Street SW, Rochester, MN 55905, USA

^*^Corresponding Author: kocher.jeanpierre@mayo.edu

## Details of Covariate Coding:

- Stage
  - Taken from any of (when available and in this order of precedence):
    clinical_stage,
    pathologic_stage,
    ajcc_pathologic_tumor_stage
  - Coded as numeric vector with values in [1,4]. E.g. all of ‘IIA’, ‘IIB’, ‘IIC’ are coded as “2”
- Grade
  - Taken from any of (when available and in this order of precedence):
    neoplasm_histologic_grade,
    tumor_grade
  - Coded as numeric vector with values in [1,4].
- Smoking Status
  - Taken from any of (when available):
    tobacco_smoking_pack_years_smoked,
    tobacco_smoking_history,
    tobacco_smoking_history_indicator
  - Coded as: never, reformed, or current
- Menopause Status
  - In BRCA, menopause_status is codified as ‘Pre’ and ‘Post’ as there are very few patients with ‘Peri’ status.
  - Patients with Peri- (the transitional stage from premenopausal to menopause) and Premenopausal status are coded as a single menopause group in this study.
- Radiation Dosage
  - Radiation dosage given to the patient translate to Gray and using estimated cumulative absorbed dose for implanted radiation sources
  - Values were either:
    - Used as-is for a continuous effect
    - Binned into 4 categories: none (≤1), low (1-5000), standard (5000-9000), and high (>9000)
- Tumor Source Site
  - Site codes are extracted from sample barcodes and used as categorical variables.
  - Sites with less than 15 samples were grouped into one category level.

## Efficacy Model Improves Pan-Cancer Models

We quantified statistical significance for a series of increasingly complex survival models, with the most complex being our pan-cancer Therapy Efficacy model. Cancer type, patient age, patient sex, tumor stage, and tumor grade accounted for much of the variance in the observed survival data. Adding a series of further clinical covariates (e.g. smoking status and others; see Table 1) significantly improved the model (likelihood ratio test: *Χ*^2^(11) = 41.3, p = 2.12x10^-5^). The addition of a random effect term for tissue source site further improved the model (*Χ*^2^(1) = 38.3, p = 6.20x10^-10^). Classifications as low- or high-risk by our Therapy Efficacy model were included and the model improvement was statistically significant (*Χ*^2^(1) = 5.4, p = 0.020). Thus, even after accounting for a wide range of clinical features often omitted from pan-cancer survival analyses, our efficacy model is a significant improvement.

In order to contextualize this improvement, we calculated the improvement that each clinical covariate provides using a leave-one-out approach. For example, inclusion of post-surgery margin status (*Χ*^2^(3) = 7.2; p = 0.066) and menopause status (*Χ*^2^(1) = 3.2; p = 0.075) each lead to modest improvements, while neither smoking status (*Χ*^2^(2) = 0.1; p = 0.930) nor lymphatic invasion (*Χ*^2^(2) = 0.8; p = 0.664) did. Thus, the variability associated with smoking stator or lymphatic invasion was already accounted for by a combination of other covariates (cancer type, patient age, sex, etc.).

## Patient Characteristics Associating with PK Model Classification

The tumor genomics features that we have considered were assessed per-patient and for the therapies that they were administered. Across all patient samples and using the Any-Hit model, the genomics features affected 557 therapy administration instances, across 389 patients. These patients were distributed across cancer types and clinical presentation (Table 2). Overall, there was an association between high-risk patients and higher tumor grade (odds ratio = 1.4, p = 1.9x10^-3^) and later tumor stage (odds ratio = 1.6, p = 1.5x10^-6^). They were also slightly younger, on average, than low-risk patients (high-risk: 57.9 years, low-risk: 61.5 years, p = 5.7x10^-4^).

5% (n = 143) patients were identified as high-risk with the therapy efficacy model. Similar trends were observed for higher grade, later stage, and younger diagnosis age.


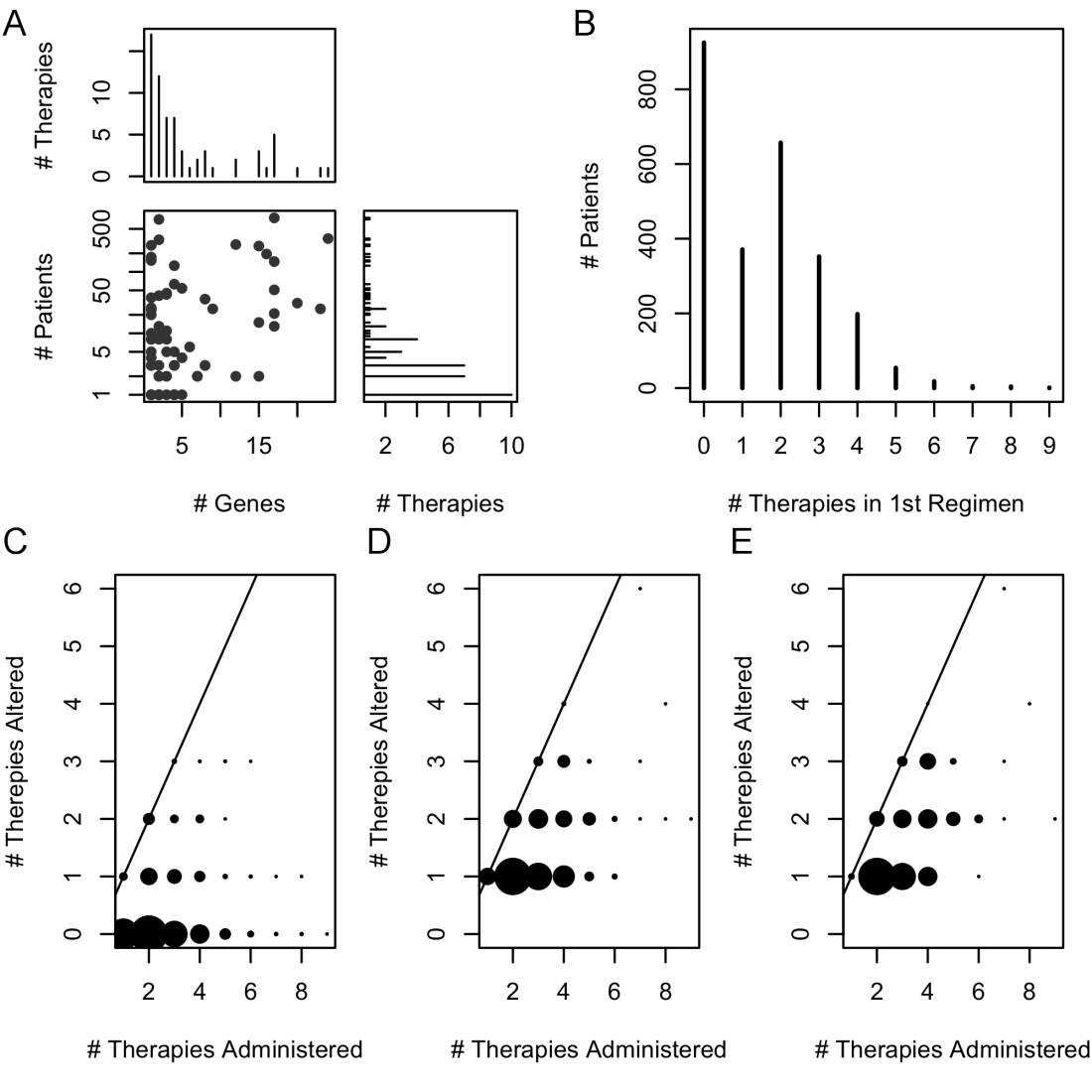


**Figure S1: Distribution and characteristics of therapies across the cohort.**

**A)** We first quantified the relationship between therapies and genes. Importantly, we manually curated gene-drug interaction data to focus on the most likely antineoplastic targets and the PK genes with greatest supporting evidence. The number of genes that we annotated as being the target of or mediating the transport or metabolism of each therapy is indicated (each point is a therapy drug). Additionally, the number of patients to which each therapy is administered is shown. Only therapies that have at least one known gene interaction are shown. All therapies have fewer than 25 gene interactions. The majority have fewer than 5. The therapies with the most interactions are broadly acting kinase inhibitors that have many potentially antineoplastic targets.

**B)** Considering all therapies regardless of whether they have known gene interactions or not, patients were most frequently administered two therapies in their first regimen. A long tail in the distribution is evident. Patients receiving seven or more therapies could indicate highly complex regimen, but more likely are those who rapidly received second line therapies but for which our semi-automated review process could not distinguish any time between therapeutic intervals.

**C)** The distribution of patients across two dimensions of therapy annotation is shown: the number of administered therapies in their first treatment regimen, and the number of those therapies identified as “altered” in their PK or target gene expression, according to the any-hit model. The plot shows the relative populations of patients in each bin. Patients who did not receive chemotherapy were not included in the plot.

**D)** We show a plot, similar to panel C, but for the sub-cohort identified as high-risk by our any-hit model (n = 389). The most common observation is a group of 116 patients receiving two therapies for which the any-hit model identified one of them with corresponding altered gene expression. Points are scaled by the number of patients falling into each bin.

**E)** We show a plot, similar to panel C, but for the sub-cohort identified as high-risk by our therapy efficacy model (n = 198).


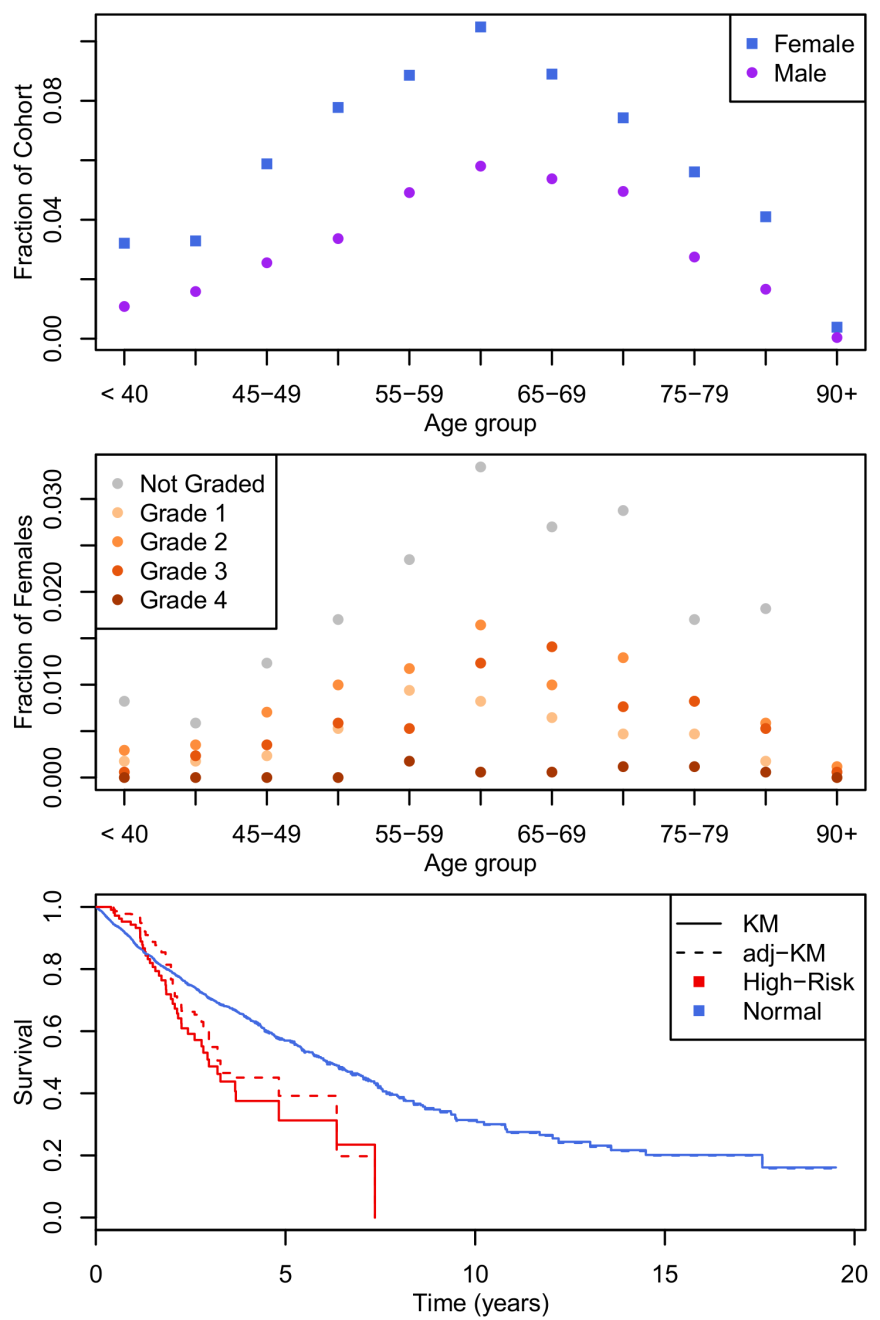

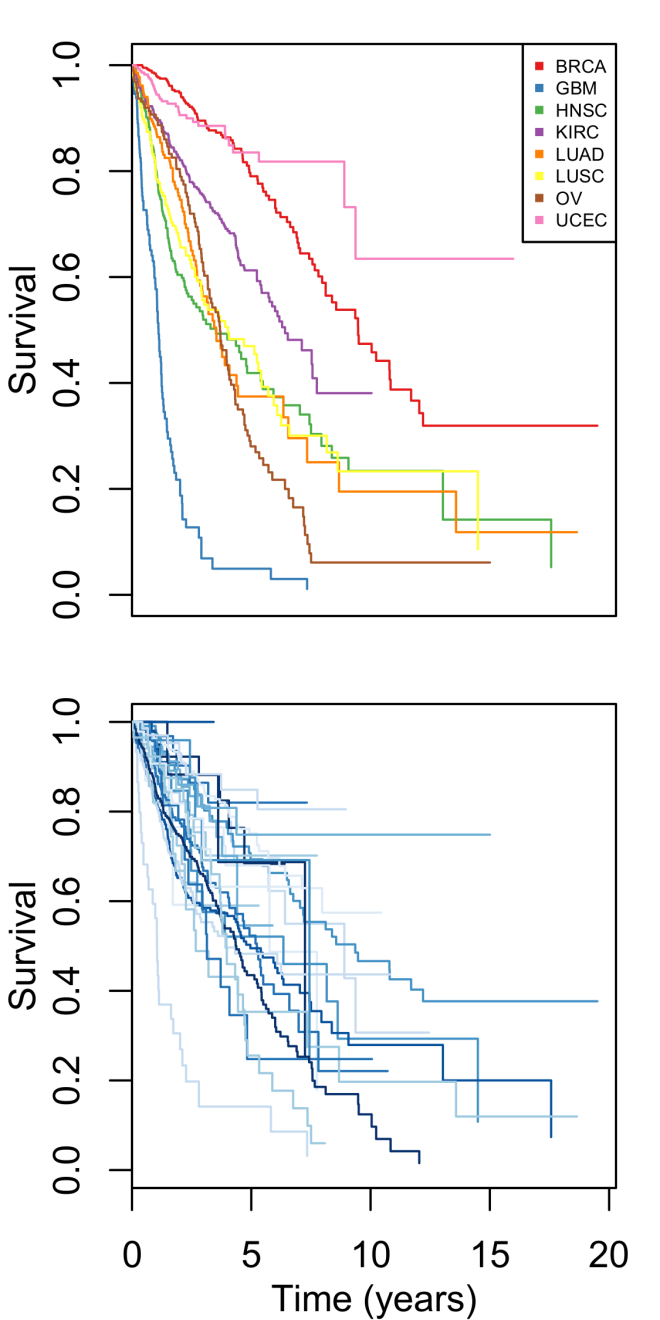


High Risk

Low Risk

**A D**

**B**

**E**

**C**

**Figure S2: Cohort characteristics.** As our study is retrospective, patients were not recruited in such a way as to balance important clinical features, as it done in prospective clinical trials. Thus, it is possible that any patient classification computed from genomics data, such as our proposed rules, are proxies for clinical features. As an attempt to remove the effect of cohort imbalance, the clinical features are added to survival models and the resulting impact of genomics scores are assessed only after the clinical covariates have been accounted for. **A)** The overall TCGA cohort has more female participants (expected due to gynecologic malignancies) that **B)** have a large range of age, grade, and other features that co-vary – the distribution of grade differs by patient age. Thus, throughout our analysis, we present adjusted and unadjusted values. **C)** The Kaplan-Meyer curves for the overall cohort separated by high expression of exporters, demonstrates a significant survival difference.

**D)** Cancer types vary in severity (each has its own intrinsic death rate) and **E)** each contributing institution may exhibit different baseline survival associations (a combination of disease type, institution specialization, patient selection, etc.), adding further complexity that we account for in order to generate more accurate models.


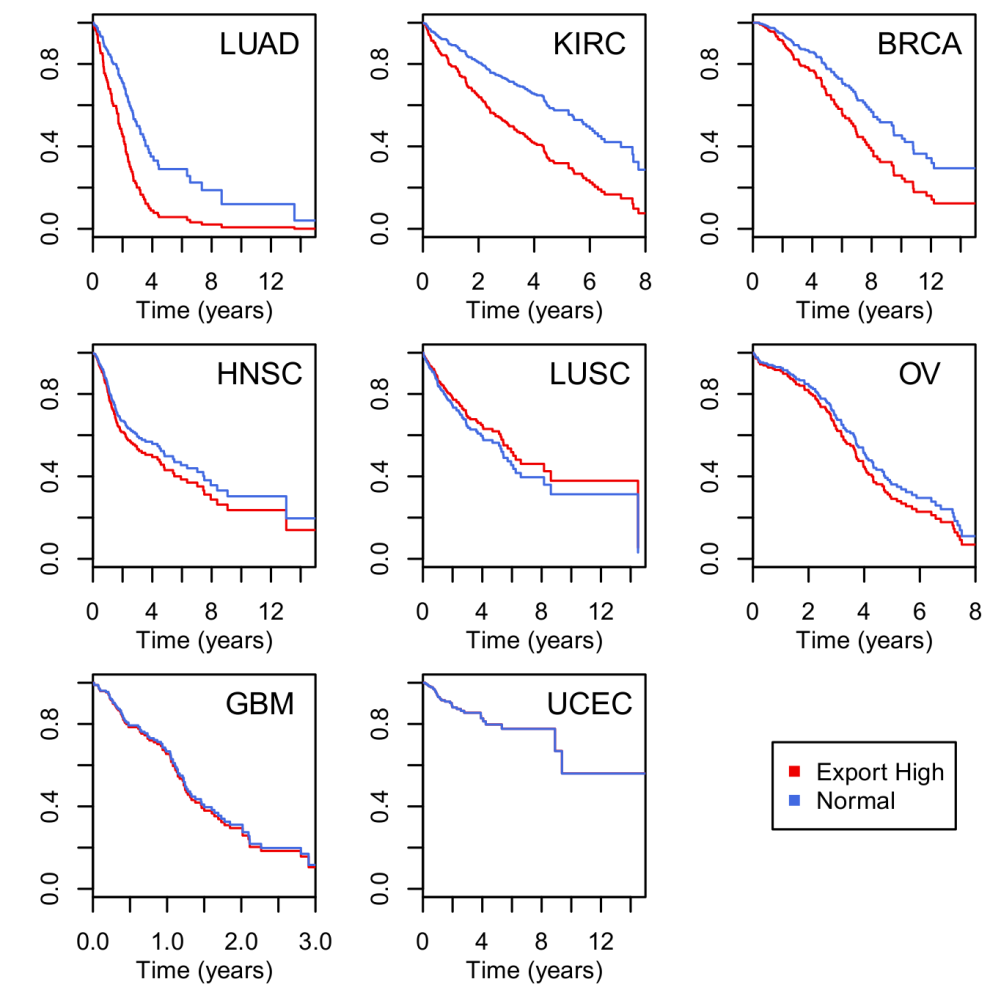
**A B**


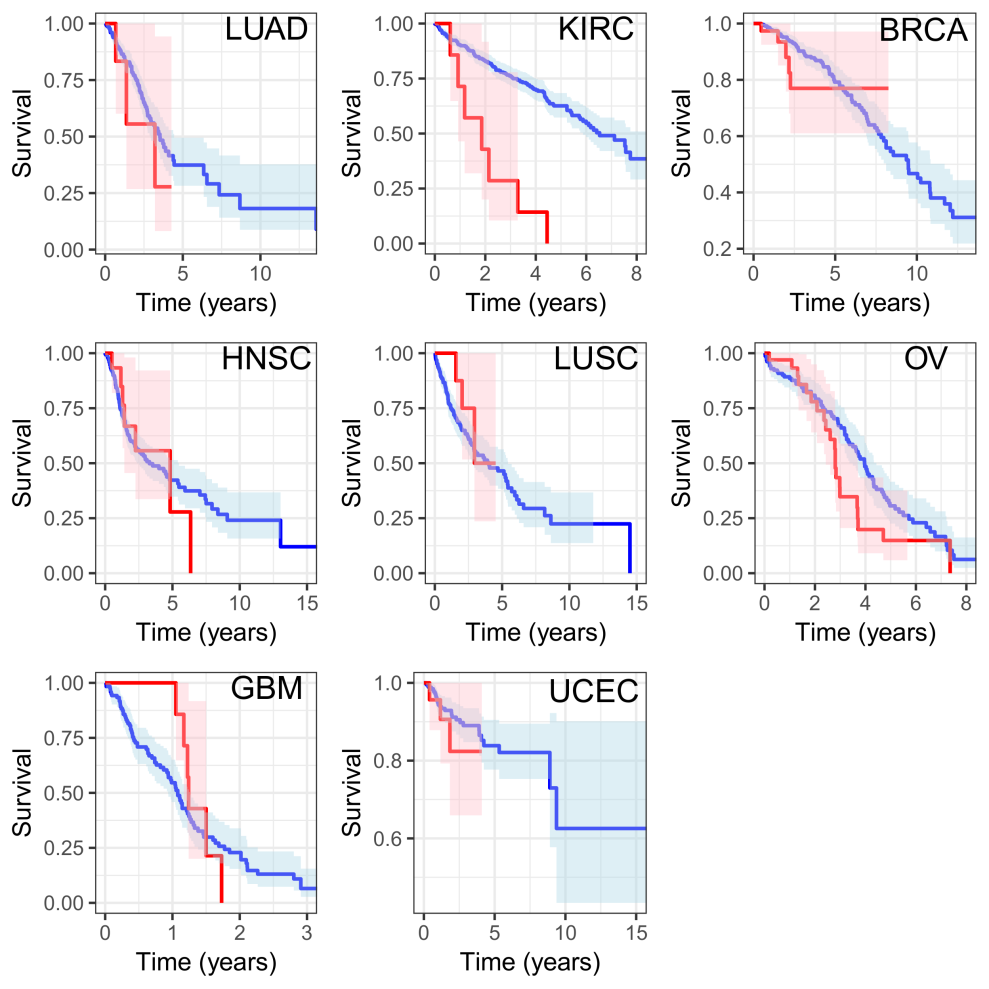

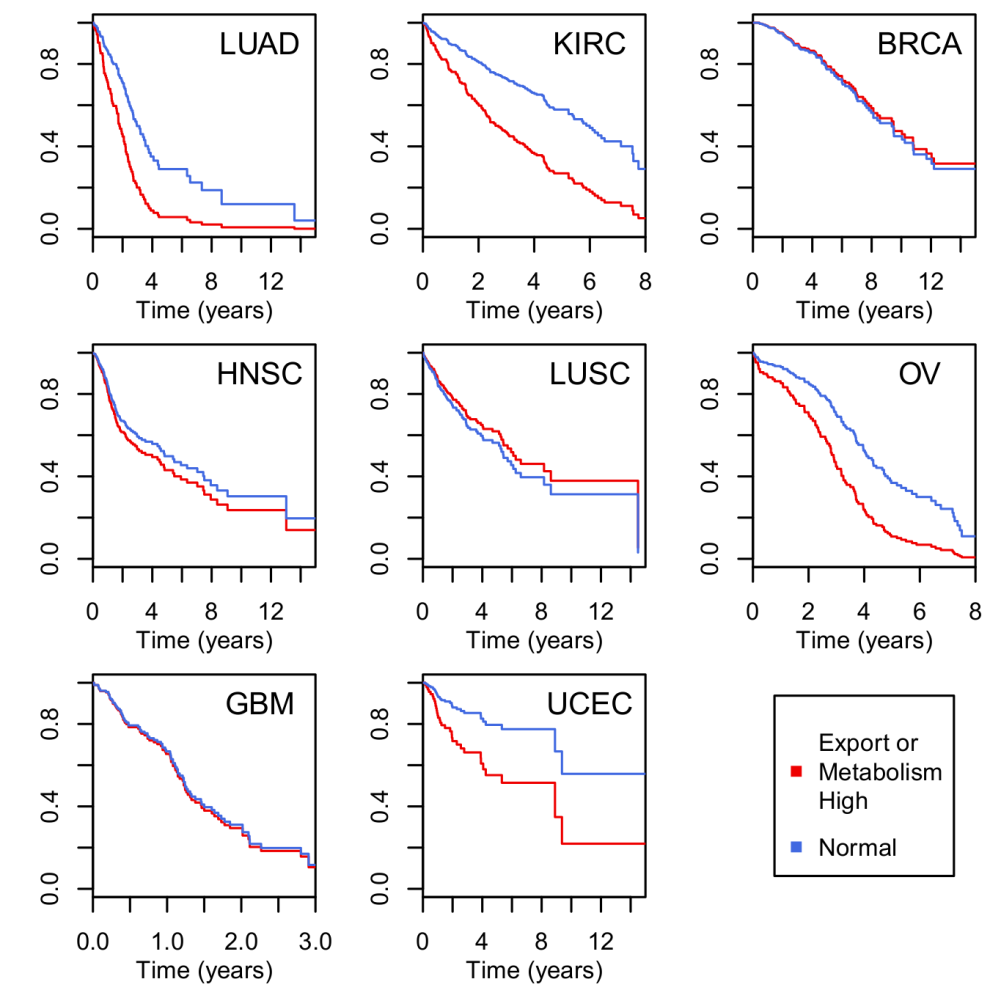


*

**Figure S3: Quantification of cancer survival implications derived from our pan-cancer models and applied per-cancer.** **A)** Similar to Figure 3, we show the survival association for the observed cohorts using our genomics-based Therapy Efficacy model. The Therapy Efficacy model leveraged two features for the administered chemotherapies: increased expression of drug export and drug metabolism genes. **B)** We include adjusted KM plots. A limitation of KM plots is their inability to represent imbalances between the two cohorts – a limitation that adjusted plots aim to address. To generate adjusted KM plots, parameters for the corresponding cancer type (model strata) are applied to predict survival curves for a standardized uniform cohort of 50 year old participants with stage-3 grade-3 disease, and either normal or high PK activity.

*For BRCA, only contributions from the exporter effect are shown. See Figure 5 and accompanying text for more details on the association between survival and metabolic gene expression in BRCA.


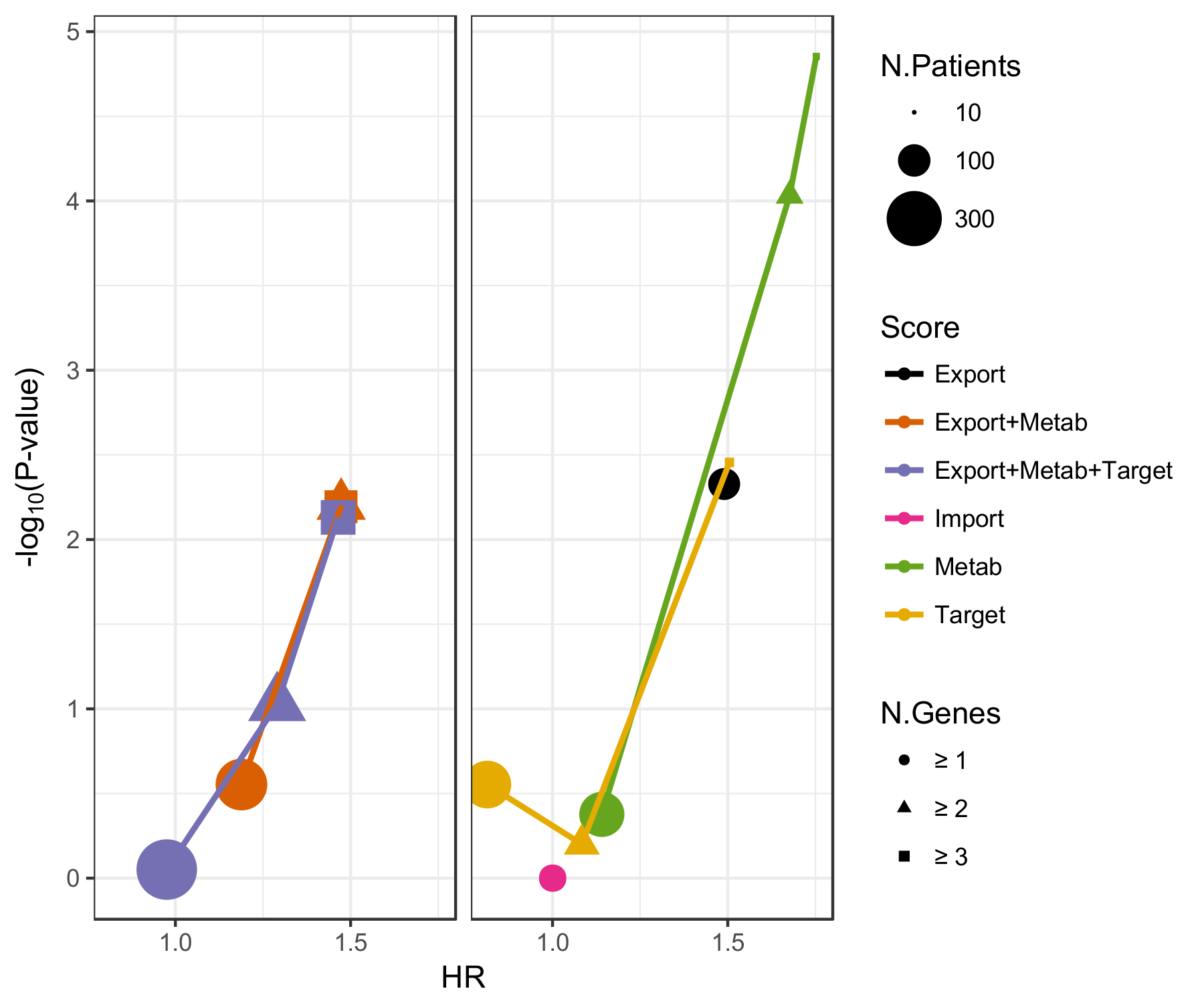


**Figure S4: Impact on survival of genomics-based rules using individual or multiple features.** We varied the number of genes (N.Genes) requiring alteration for each rule (i.e. if ≥ 1 or ≥ 2 target genes need to be expressed at Z < -2 for us to call the therapy’s efficacy impaired). As the stringency required for any rule increases, the number of patient’s (N.Patients) that it will apply to decreases, while the biologic effect size increases (more significant HR).


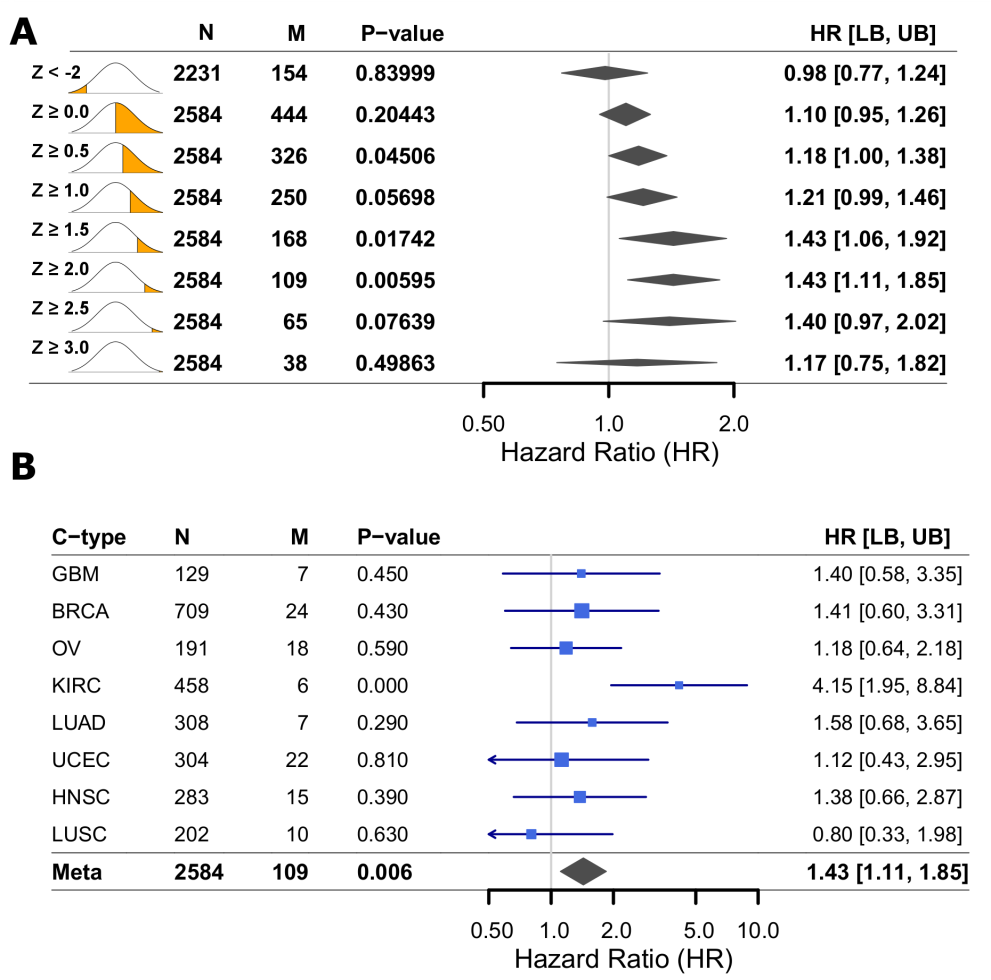


**Figure S5: Association between drug exporter gene status and event-free survival. A)** We show the results of our meta-analysis across tumor types and for different levels of expression threshold. Moderate increases in expression identify larger numbers of patients (M) affected, but at a lower level of increased risk of disease-related events, whereas more stringent thresholds identify a smaller number of patients at increased risk. **B)** For the threshold Z ≥ 2, we show the forest plot of per-cancer associations from which the meta-analysis is performed. While few individual cancer types exhibit independently statistically significant associations, effects are consistently hazardous as summarized in the meta-analysis.


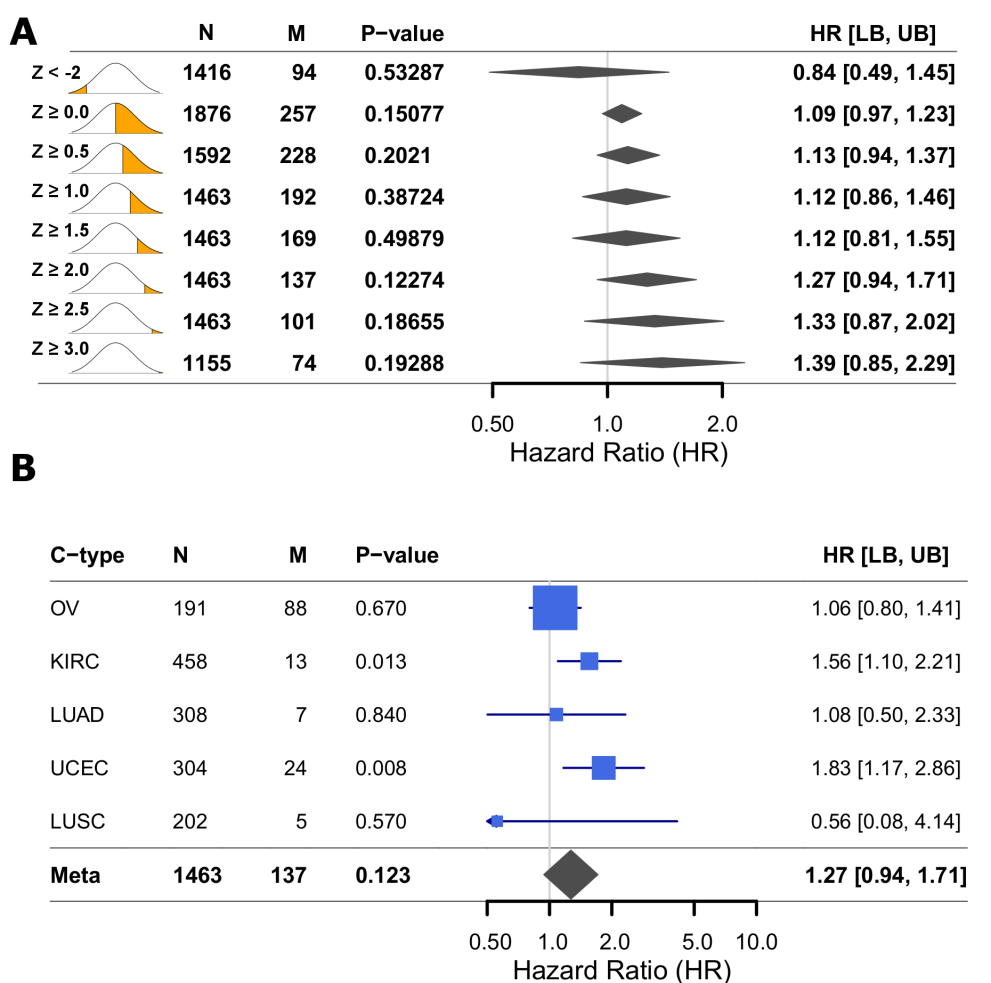


**Figure S6: Increased expression of drug metabolism genes for administered therapies conveys increased risk of cancer mortality.** **A)** Results are consistent with prior expectation from the literature, but are not independently significant in meta-analysis. BRCA demonstrated an opposite association and was excluded from this analysis. Date presented as in Figure 4. **B)** Forest plot for Z ≥ 2.


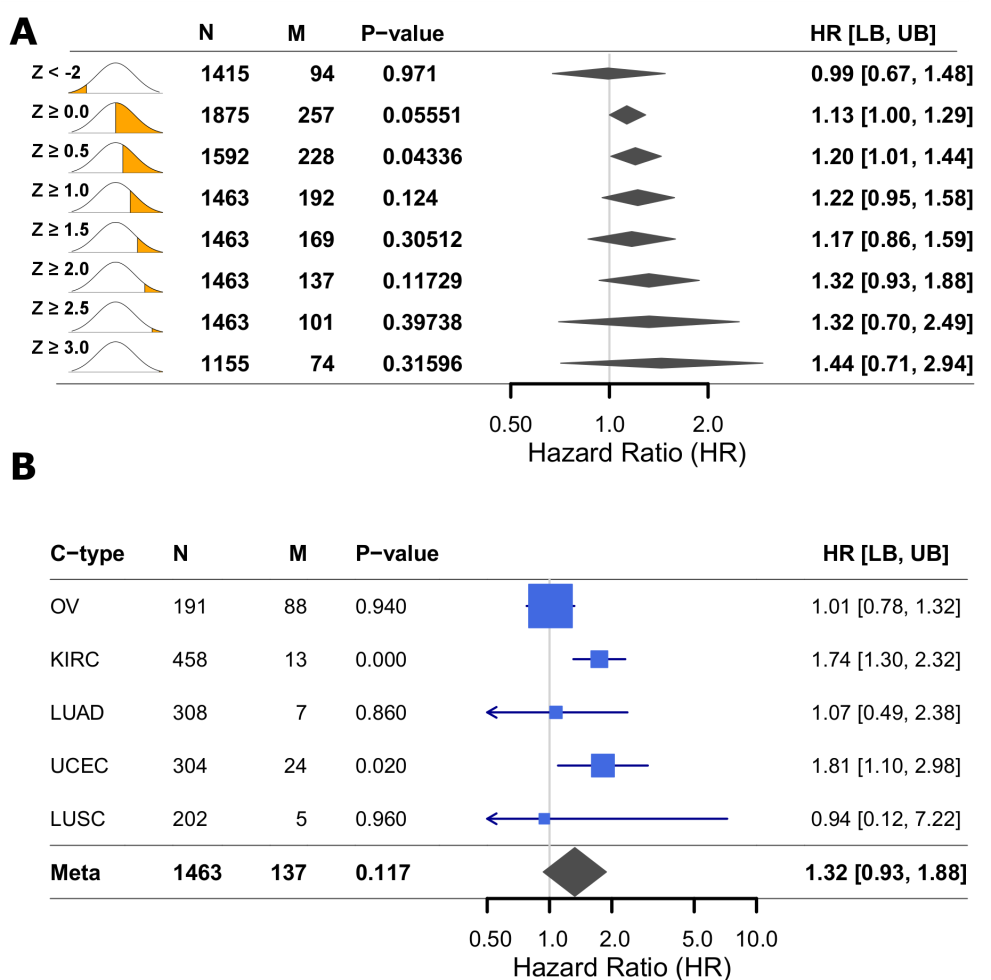
 Z ≥ 2.0

**C**


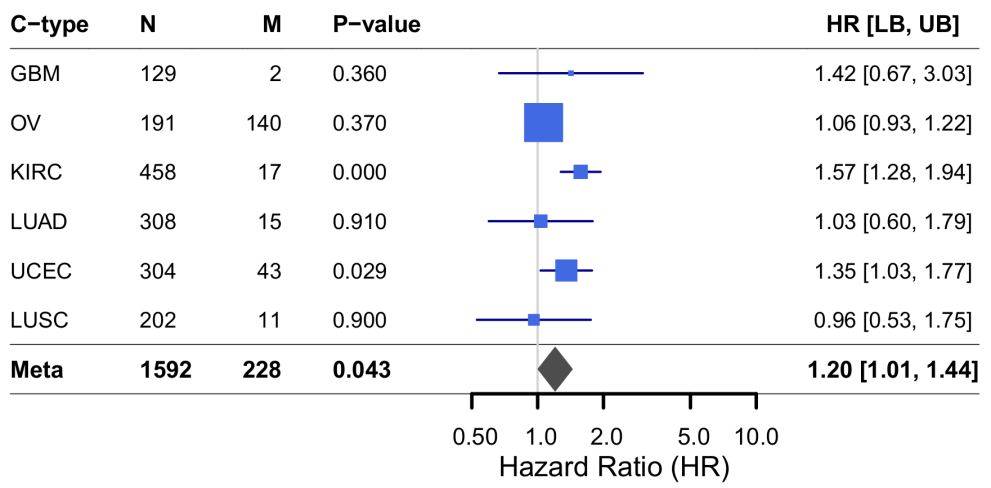
 Z ≥ 0.5

**Figure S7: Association between drug metabolism gene status and event-free survival. A)** Our meta-analysis is summarized similar to previous analyses and the per-cancer associations for the **B)** threshold Z ≥ 2 and **C)** Z ≥ 0.5 thresholds shown. The meta-analysis of drug metabolism effects demonstrates a less smooth progression with increasing Z-score threshold due largely to drop-out of cancer types; some cancer types have no samples exhibiting higher levels of metabolism-gene activation, leading to their exclusion from the meta-analysis.


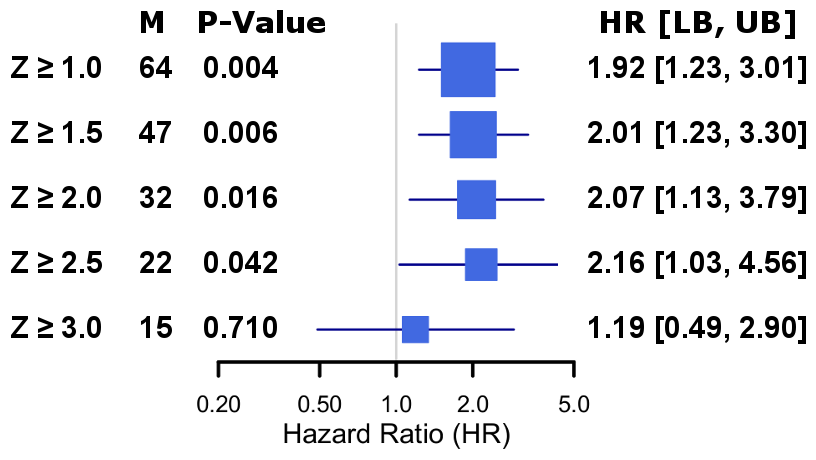


**Figure S8: HMGB1 association in OV.** After filtering patients with increased exporter expression or decreased target expression, for their administered therapies, we computed association between survival and HMGB1 expression for OV patients receiving platinum-based therapies (n=219). High HMGB1 expression is associated with poorer outcomes at all elevated Z-score thresholds and with comparable effect sizes. As in previous figures, M represents the number of patient samples with expression level above the indicated Z-score threshold.
